# Absorption and fixation times for evolutionary processes on graphs

**DOI:** 10.1098/rsos.260131

**Published:** 2026-11-11

**Authors:** Fernando Alcalde Cuesta, Gustavo Guerberoff, Álvaro Lozano Rojo

**Affiliations:** ^1^ Facultad de Matemáticas, Universidad de Santiago de Compostela, Santiago de Compostela, Spain; ^2^ Instituto de Matemática y Estadística Rafael Laguardia, Facultad de Ingeniería, Universidad de la República, Montevideo, Uruguay; ^3^ Departamento de Matemáticas, Universidad de Zaragoza, Zaragoza, Spain

**Keywords:** evolutionary dynamics, Moran and proliferation processes, absorption and fixation times

## Abstract

In this paper, we study the absorption and fixation times for evolutionary processes on graphs, with relative fitness r>0, under different updating rules. While in the Moran process a single neighbour is randomly chosen to be replaced, in proliferation processes, other neighbours can be replaced using Bernoulli or binomial draws depending on 0<p≤1. There is a critical value $p_{c}$ such that the proliferation is advantageous or disadvantageous in terms of fixation probability depending on whether $p>p_{c}$ or $p<p_{c}$. We clarify the role of symmetries for computing the fixation time in the Moran process. We show that the Maruyama–Kimura symmetry depends on the graph structure induced in each state, implying asymmetry for all graphs except cliques and cycles. There is a fitness value, not necessarily 1, beyond which the fixation time decreases monotonically. We apply Harris’s graphical method to prove that the fixation time decreases monotonically depending on p. Thus, there exists another value $p_{t}$ for which the proliferation is advantageous or disadvantageous in terms of time. However, at the critical level $p=p_{c}$, the proliferation is highly advantageous when r tends to infinity.

## 1. Introduction

Proliferation models were introduced in [1] to more realistically describe the propagation of mutant individuals in a finite population arranged on a graph. Resident individuals evolve like in the structured version [2] of the classical Moran process [3], that is, if the individual randomly chosen for reproduction is a resident, or *binomial* then its clone replaces a single neighbour randomly chosen for death. However, if the individual chosen for reproduction is a mutant, we distinguish two types of draw: *Bernoulli*, where all or none of the neighbours become mutants depending on a certain probability p, where the number of neighbours chosen for death follows a binomial distribution with probabilistic parameter p; or *binomial*, where the number of neighbours chosen for death follows a binomial distribution with probabilistic parameter p. Both processes were used to model the spread of prion diseases and the proliferation of stem cells that produce multiple daughter cells, see [1] and references therein.

The success of the spread of stem cells or prions depends on the rate at which they physically divide or spread, as measured by the parameter p. However, although it may seem surprising, proliferation is not always to the advantage of mutants. The main result of [1] states that, whatever the underlying population structure, there always exists a critical value $p_{c}$ (depending on the graph structure) such that the fixation probability for the proliferation process is equal to the fixation probability for the corresponding Moran process. If $p>p_{c}$, proliferation is advantageous, but if $p<p_{c}$, it is disadvantageous. Symbolic computation for a small population shows that this critical value is highly correlated to the mean degree of the graph so that proliferation is more advantageous in cliques than in star graphs. By random updating of the proliferation parameter, fluctuating waves in the population of proliferating mutant individuals (similar to the *parasitaemia waves* exhibited in [4]) can be also created, replacing fixation or extinction by a quasi-stationary state.

In this paper, we are interested in the absorption and fixation times. The *mean absorption time* is the mean number of steps needed to reach one of the two absorbing states of the process, namely, fixation or extinction of mutants, starting from any mutant or any set with the same number of mutants. The *mean fixation time* is the mean time required for any mutant (or any set with the same number of mutants) to take over the whole population, assuming that this will happen.

Similar to fixation probability, definitions and derivations of these times for birth–death processes on (infinite) homogeneous populations can be found in [5, ch.4, §7]. A subsequent description of finite-state continuous-time Markov chains [5, ch.4, §8] was later used by Ashcroft *et al.* [6] and independently by Hathcock & Strogatz [7] to study the distribution of mean fixation times in a finite homogeneous population. A full analytic computation was given by Antal & Scheuring [8] in the homogeneous case, whereas computations for the Moran process on complete, cycle and star graphs were derived in [9,10].

Works by Maruyama [11] and Maruyama & Kimura [12] in the 1970s were the first to observe a symmetry between the fixation time of a set of i mutants and the extinction time of the complementary set of N−i residents in a homogeneous population. This symmetry was later rediscovered by Ashcroft *et al.* [6] and also Taylor *et al.* [13] on average. More recently Hathcock & Strogatz [7] derived that the fixation time distributions of mutants of fitness r and 1/r are identical in this case. We obtain both symmetries from another kind of symmetry in a clique, which we call *G-symmetry*, and we use it to study the monotonicity of the fixation time of any mutant in the Moran process on any graph. Although one would expect this time to decrease if the mutant is advantageous and his advantage increases, we shall see that this is not strictly true.

Similarly, it could be expected that proliferation might reduce the mean fixation time, at least if the fixation probability were identical for both processes. Here, we indeed prove that, in the critical regime, proliferation of mutant individuals reduces the mean absorption and fixation times, at least for some large classes of graphs. However, we also show the existence of a new critical probability $p_{t}$ determining the transition from the regime where proliferation does not reduce the fixation time to the regime where proliferation reduces the fixation time.

This dual objective determines the structure and methods used in the paper. In the first part, both absorption and fixation times are explicitly calculated for complete, cycle and star graphs by solving non-homogeneous linear systems associated with discrete-time Markov chains, as in the algebraic approach used in the non-proliferating case [8–10]. Poisson clocks and Harris’s graphical model were previously used in [1] to prove that fixation probability of mutants, like stem cell or prions, depends monotonically on the physical rate of their division or propagation, according to the parameter p. However, although various approaches have been tried, it did not seem straightforward to apply these methods to adapt this result to the fixation time of mutants for all graph structures. Ultimately, it was the explicit calculations in the first part that led to the idea of introducing a ‘non-backward’ process, which we have termed the ‘red process’, and to providing indirect evidence of monotonicity. As invasion processes, Bernoulli and binomial proliferation processes have been basically compared to the Moran process, in which the dependence of the fixation time on the relative fitness is much more subtle, as shown in [6,7], and the Maruyama–Kimura symmetry becomes fundamental. This creates a certain contrast between the two parts of the article, which have different perspectives and approaches. This also led us to focus on invasion processes and to defer the comparison of proliferation rules with other relevant updating rules, such as ‘death–birth’ (DB), ‘imitation’ (IM) or ‘pairwise comparison’ (PC), related to the voter model (see [14,15] and references therein).

More precisely, in §3, we explicitly compute absorption and fixation times for complete, cycle and star graphs of order N for Bernoulli proliferation, allowing to compare these times to the corresponding times for the Moran process on each graph. The critical case where the proliferation is neither advantageous nor disadvantageous is particularly interesting. In all studied cases, this critical rate (Moran times/proliferation times, at $p=p_{c}$) is strictly greater than one for any relative fitness value r>0. The limit of the critical rate coincides for both, absorption and fixation times, as r→+∞, but depending on the type of graph, this limit is equal to

— Complete graph: $(N-1)H_{N-1}$, where $H_{N-1}$ is the (N−1)-harmonic number,
— Cycle graph: $\frac{2N(N-1)}{\left(N+1)(N-2\right)}$, and
— Star graph: $\frac{N}{3N-2}\left[(N-1)\sum_{i=1}^{N-1}H_{i}\right]$.

Analytical expressions and rate limits are completed with the exact computation of times and rates for all graphs of order 6 included in S4 in the supplementary material. Both approaches confirm that mean absorption and fixation proliferation times are lower for star and complete graphs, with cycles being the worst performing graphs.

In §4, we also give complete derivations of mean absorption times for binomial proliferating and non-proliferating individuals on a complete graph. Absorption and fixation times and rates are graphically described for all graphs of order 6 and also for some additional orders, see in S4 and S5 in the supplementary material.

Finally, in §§5 and 6, we demonstrate the existence of a second critical value $p_{t}$ such that the fixation time rate (i.e. the ratio of the Moran fixation time to the proliferation fixation time for a fixed r) is greater or less than 1 depending on whether p is greater or less than $p_{t}$. This implies the existence of three different regimes when we compare Bernoulli or binomial proliferation with the classical Moran process: chance and time disadvantageous, chance or time disadvantageous, and finally chance and time advantageous. As explained above, the methods used in these final sections are entirely different from the algebraic methods employed in the first part. Certain theoretical aspects relating to this second part, specifically *embedded jump process*, *Doob’s transform*, *Harris’s graphical model* and the *red process*, are discussed in S2 and S3 in the supplementary material.

## 2. Proliferation models on evolutionary graphs

*Proliferation models* on finite graphs G=(V,E) were introduced in [1]. All such graphs are assumed connected and undirected. As for the Moran model [3] and its structured version [2], see also [16–22], we assume the following:

— A finite population is arranged on the vertex set V of cardinal |V|=N.
— There exist two types of individuals/particles/alleles/cells: *mutant/resident*, *type-1/type-2*, *advantageous/disadvantageous* or *mutant/wild-type*.
— Mutant individuals have a relative fitness r>0 with respect to resident individuals.
— The population evolves when clones of a mutant or resident individual occupying a vertex v replace individuals, called *neighbours*, occupying vertices w linked to v by an element of the edge set E.

To define the evolutionary process, we suppose that, at a given time n, there are i mutants and N−i residents occupying V. Let $S_{n}\subset V$ be the set of vertices occupied by mutants at this time. A vertex v is randomly chosen with probability $p_{v}=\frac{r}{w_{i}}$ if $v\in S_{n}$, and $p_{v}=\frac{1}{w_{i}}$ if $v\notin S_{n}$, where $w_{i}=ri+N-i$ is the total reproductive weight of all individuals at this state. As in the Moran process, if the vertex v is occupied by a resident then we randomly choose a neighbour w of v and put a resident on the vertex w. If v is occupied by a mutant, under the classical model, one of its neighbours w is chosen uniformly at random and becomes occupied by a mutant. However, in [1], we considered two types of clonal proliferation, both associated with a parameter p∈[0,1] which regulates its intensity:

— **Bernoulli proliferation**: with probability p, all the neighbours of vertex v (whose number d(v) is the *degree of v*) become occupied by mutants, and with probability 1−p, nothing happens.
— **Binomial proliferation**: each neighbour of v is occupied by a mutant with probability p independently. Thus, the number of potentially occupied neighbours of v follows a binomial distribution with parameters d(v) and p.

The *fixation probability* is the basic quantity for the stochastic analysis of the proliferation studied in [1]. For the Moran process on a graph G=(V,E), given a set of vertices S⊂V occupied by mutants, the *fixation probability*

$$\Phi_{S}^{M}(r)=P\;[\;\exists n\geq 0:S_{n}=V|S_{0}=S\;]$$

is the solution of a system of $2^{N}$ linear equations with boundary conditions $\Phi_{\emptyset }^{M}(r)=0$ and $\Phi_{V}^{M}(r)=1$. Here, $S_{n}$ denotes the set of vertices occupied by mutants at time n≥0. As G is undirected, such a linear system admits a unique solution [23]. The *(mean) fixation probability* is given by

$$\Phi_{1}^{M}(r)=\frac{1}{N}\sum_{v\in V}\Phi_{\left\{v\right\}}^{M}(r),$$

being a rational function on the fitness r. Similarly, we denote by $\Phi_{1}^{B}(r,p)$ and $\Phi_{1}^{b}(r,p)$ the mean fixation probability for Bernoulli and binomial proliferation on G with fitness r and proliferation parameter p, respectively. In [1], we compared the fixation probabilities for proliferating and non-proliferating processes obtaining the following result.

**Theorem 2.1.**

*[1] For any undirected graph G, there is a unique pair of critical values $p_{c}^{B}=p_{c}^{B}(r)$ and $p_{c}^{b}=p_{c}^{b}(r)$ depending on the fitness r>0 such that*

$$\Phi_{1}^{B}(r,p_{c}^{B})=\Phi_{1}^{M}(r)\;and\;\Phi_{1}^{b}(r,p_{c}^{b})=\Phi_{1}^{M}(r).$$

Thus, for every fitness value r>0, there is a transition between two different regimes where the proliferation is *advantageous* for mutants if $p>p_{c}^{\beta }(r)$, but *disadvantageous* if $p<p_{c}^{\beta }(r)$, with β=b,B. As explained in [1], even assuming that the parameter p is critical, equal to $p_{c}^{\beta }$, proliferation alters the classification into amplifiers and suppressors with respect to the Moran process [14,24–26].

Here, we study other fundamental quantities in the stochastic analysis of the evolution in a finite population: the *mean absorption time* and the *mean fixation time*. Denoting again $S_{n}$ the set of vertices occupied by mutants at time n≥0, for each subset S of V, the *absorption time* is the random variable

$$\tau_{S}^{\beta }=\min\{\;n\geq 0\;|\;S_{n}=\emptyset \text{ or }S_{n}=V\;\}$$

conditioned to $S_{0}=S$. Its expectation is given as solution of a system of linear equations, although this is now non-homogeneous. With a slight abuse of notation, we denote both the random variable and its expectation by the same symbol. The *mean absorption time* $\tau_{i}^{\beta }$ is the average of the expected times $\tau_{S}^{\beta }$ associated with the subsets S having i elements. In the next sections, we consider the equations in some specific cases, although a precise general description is given in S1 in the supplementary material. The *fixation time* $T_{S}^{\beta }$ is the expectation of the random variable $\tau_{S}^{\beta }$, but conditioned to the fixation of mutants (see also supplementary material, S1). The *mean fixation time* $T_{i}^{\beta }$ is again the average of $T_{S}^{\beta }$ when |S|=i. For convenience, we usually omit the term mean for the times $\tau_{i}^{\beta }$ and $T_{i}^{\beta }$.

For comparing the absorption and fixation times $\tau_{1}^{\beta }$ and $T_{1}^{\beta }$ of a single proliferating mutant (on average) to the absorption and fixation times $\tau_{1}^{M}$ and $T_{1}^{M}$ of a single non-proliferating mutant in the usual Moran process, we introduce the *absorption* and *fixation rates*

$$\rho^{\beta }(r,p)=\frac{\tau_{1}^{M}(r)}{\tau_{1}^{\beta }(r,p)}\;\text{and}\;{\text{P}}^{\beta }(r,p)=\frac{T_{1}^{M}(r)}{T_{1}^{\beta }(r,p)}.$$

We are especially interested in the case $p=p_{c}^{\beta }(r)$ where proliferation does not imply an advantage or a disadvantage in respect to the fixation probability, see §§3 and 4. The indices M and β are suppressed when the type of process is clear. At the limit when r→+∞, we can observe the following properties (which are proved in the supplementary material, S2):

**Proposition 2.1.**

*For any graph G, for any state S≠∅,V, and for any p>0, we have*

$$\lim_{r\to +\infty }\Phi_{S}^{M}(r)=\lim_{r\to +\infty }\Phi_{S}^{\beta }(r,p)=1,$$

*where β∈{b,B}. We also have*

$$\lim_{r\to +\infty }\tau_{S}^{M}(r)=\lim_{r\to +\infty }T_{S}^{M}(r)\;and\;\lim_{r\to +\infty }\tau_{S}^{\beta }(r,p)=\lim_{r\to +\infty }T_{S}^{\beta }(r,p).$$

These quantities will be later interpreted as the fixation time of the so-called *red process* associated with the Moran process or the proliferation processes, see §5 and S3 in the supplementary material.

As with the fixation probability, it is natural to be interested in the monotonicity of the mean absorption and fixation times for all processes considered here. Figure 1 shows the difference between unconditional and conditional times for the Moran process.

**Figure 1.**
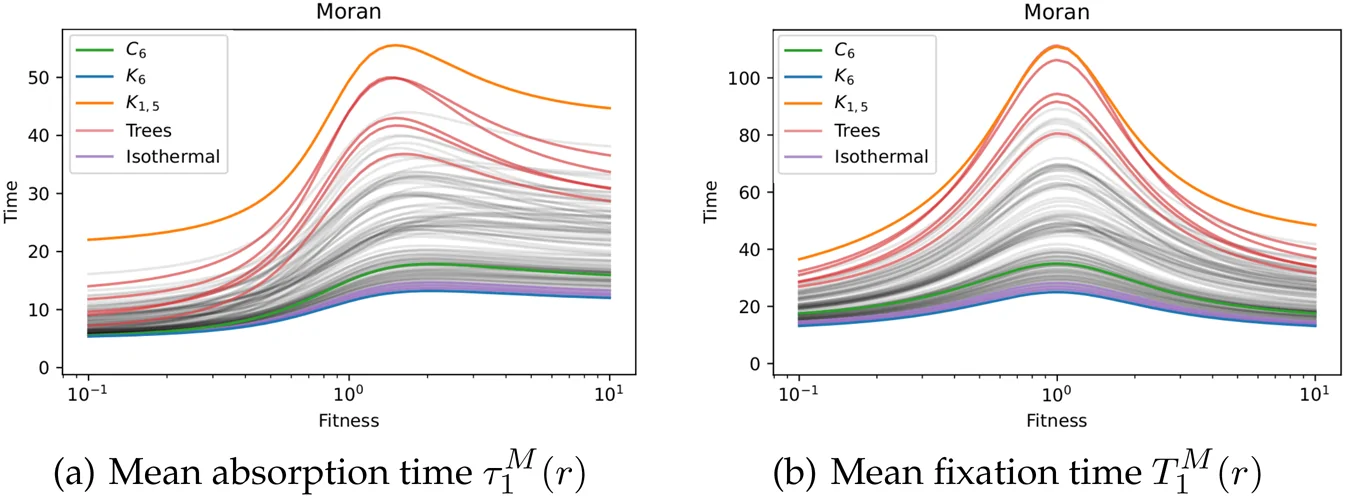
(a) Mean absorption time and (b) mean fixation time for the Moran process on all graphs of order 6.

As we shall see in §5, the symmetry of expected fixation times is only apparent, unless the graph is a clique or a cycle. The following theorem details the general situation.

**Theorem 2.2.**

*For any graph G and for any vertex v∈V, there exist a fitness value r=r(v)>0 depending on v such that the fixation time $T_{\left\{v\right\}}^{M}$ decreases on [r,+∞).*

For proliferation processes, we prove the following monotonicity result.

**Theorem 2.3.**

*For any graph G, for any fitness value r>0, and for any vertex v∈V, we have that $T_{\left\{v\right\}}^{\beta }(r,p^{\prime })\geq T_{\left\{v\right\}}^{\beta }(r,p)$ if $0<p^{\prime }\leq p$.*

From this result, we deduce the existence of a second phase transition $p_{t}$ for the mean fixation time of proliferating mutant individuals.

**Theorem 2.4.**

*For any graph G and for any fitness value r>0, there exists a proliferation parameter $p_{t}^{\beta }=p_{t}^{\beta }(r)$ such that ${\text{P}}^{\beta }(r,p)\leq 1$ if $p\leq p_{t}^{\beta }$ and ${\text{P}}^{\beta }(r,p)\geq 1$ if $p\geq p_{t}^{\beta }$.*

## 3. Absorption and fixation times for Bernoulli proliferation

In this section, we describe the absorption and fixation times for Bernoulli proliferation on cliques, cycles and star graphs. The method we use is quite similar to that used in [9,10] to compute the absorption and fixation times for the Moran process on graphs, although Bernoulli proliferation simplifies some computations.

### 3.1. Absorption time for Bernoulli proliferation on complete graphs

Let $G=K_{N}$ be the complete graph of order N. Since $K_{N}$ is symmetric, if the starting state S contains i mutants, we can assume S={1,…,i} as in figure 2a. Then, S can only evolve to a state with i−1 mutants (equal to {1,…,i−1} up to symmetry), S or V. The non-trivial transition probabilities are given by

(3.1) $$\begin{array}{rl} p_{i,i-1} & =\frac{N-i}{w_{i}}\frac{i}{N-1},\\ p_{i,N} & =\frac{rpi}{w_{i}},\\ \text{and }p_{i,i} & =1-p_{i,i-1}-p_{i,N}. \end{array}$$

For i=1,2,…,N−1, the absorption times $\tau_{i}$ satisfy the equation

(3.2) $$\begin{array}{r} \begin{array}{rl} \tau_{i} & =p_{i,i-1}\tau_{i-1}+p_{i,N}\tau_{N}+(1-p_{i,i-1}-p_{i,N})\tau_{i}+1\\ & =p_{i,i-1}\tau_{i-1}+(1-p_{i,i-1}-p_{i,N})\tau_{i}+1 \end{array} \end{array}$$

as $\tau_{N}=0$, see figure 2b. Thus, we get

(3.3) $$\begin{array}{r} \begin{array}{rl} \tau_{i} & =\frac{p_{i,i-1}}{p_{i,i-1}+p_{i,N}}\tau_{i-1}+\frac{1}{p_{i,i-1}+p_{i,N}}\\ & =\frac{1-\frac{i-1}{N-1}}{1+rp-\frac{i-1}{N-1}}\tau_{i-1}+\frac{1}{1+rp-\frac{i-1}{N-1}}\frac{w_{i}}{i}. \end{array} \end{array}$$

**Figure 2.**
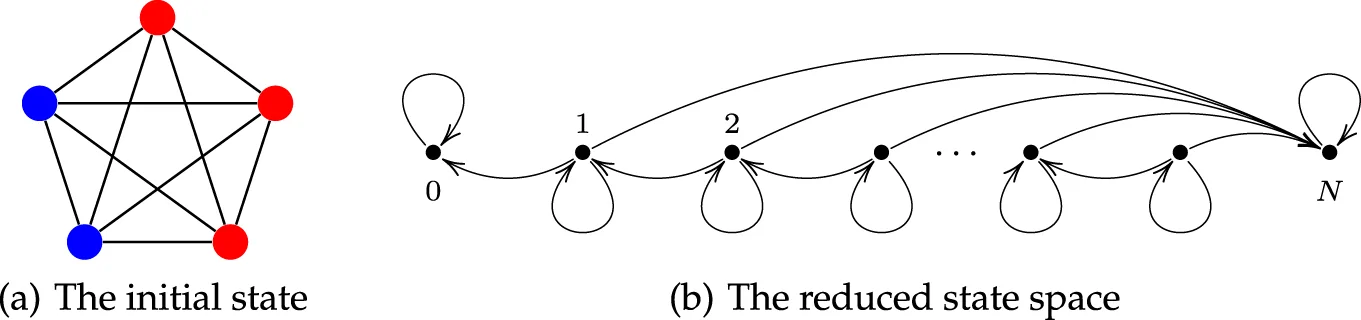
Bernoulli proliferation on a complete graph: (a) the initial state S={1,2,3} and (b) the reduced state space.

Reasoning by recurrence, for i=1,2,…,N−1, we deduce

$$\begin{array}{rl} \tau_{1} & =\frac{w_{1}}{1+rp}\\ \text{and }\tau_{2} & =\frac{1-\frac{1}{N-1}}{1+rp-\frac{1}{N-1}}\frac{w_{1}}{1+rp}+\frac{1}{1+rp-\frac{1}{N-1}}\frac{w_{2}}{2}, \end{array}$$

and in general

(3.4) $$\tau_{i}=\sum_{j=0}^{i-1}\frac{1}{\left[1+rp-\frac{j}{N-1}\right]}\frac{w_{j+1}}{j+1}\prod_{k=j+1}^{i-1}\frac{1-\frac{k}{N-1}}{1+rp-\frac{k}{N-1}},$$

where the empty product (for j=i−1) is equal to 1.

This time can be compared with the absorption time for the Moran process on the same graph [9,10]:

(3.5) $$\tau_{i}^{M}=\Phi_{i}^{M}\sum_{j=1}^{N-1}\frac{1}{p_{j,j+1}^{M}}\sum_{k=j}^{N-1}\prod_{l=j+1}^{k}\gamma_{l}-\sum_{j=1}^{i-1}\frac{1}{p_{j,j+1}^{M}}\sum_{k=j}^{i-1}\prod_{l=j+1}^{k}\gamma_{l},$$

where the empty sum (for i=1) is equal to 0. Here, $p_{j,j+1}^{M}=\frac{rj}{w_{j}}\frac{N-j}{N-1}$ for all j=1,…,N−1 and $\gamma_{l}=1/r$ for all l=1,…,N−1.The fixation probability for the Moran process is given by

(3.6) $$\Phi_{i}^{M}=\frac{1+\sum_{j=1}^{i-1}\prod_{k=1}^{j}\gamma_{k}}{1+\sum_{j=1}^{N-1}\prod_{k=1}^{j}\gamma_{k}}=\frac{1-\frac{1}{r^{i}}}{1-\frac{1}{r^{N}}}=r^{N-i}\frac{r^{i}-1}{r^{N}-1}.$$

So that we have

(3.7) $$\begin{array}{r} \begin{array}{rl} \tau_{i}^{M}= & \;r^{N-i}\frac{r^{i}-1}{r^{N}-1}\left(\sum_{j=1}^{N-1}\frac{1}{p_{j,j+1}^{M}}\frac{1}{r^{N-j-1}}\frac{r^{N-j}-1}{r-1}\right)\\ & -\sum_{j=1}^{i-1}\frac{1}{p_{j,j+1}^{M}}\frac{1}{r^{i-j-1}}\frac{r^{i-j}-1}{r-1}, \end{array} \end{array}$$

where in the last term the empty sum (for i=1) remains equal to 0. In particular, the *absorption time rate* is

(3.8) ρ(r,p)=τ1Mτ1 =rN−1r−1rN−1(∑j=1N−1wjrjN−1N−j1rN−j−1rN−j−1r−1)r+N−11+rp=(1+rp)N−1r+N−1(∑j=1N−1rj+N−jrj(N−j)rN−rjrN−1o),

and more precisely

(3.9) ρ(r,pc)=rN−1+⋯+r+1rN−2+⋯+r+1N−1r+N−1(∑j=1N−1rj+N−jrj(N−j)rN−rjrN−1o),

when p is equal to the critical value,

$$p_{c}=\frac{r^{N-2}}{r^{N-2}+\cdots +r+1},$$

described in [1]. Although in this case, where proliferation does not imply an advantage or a disadvantage for the fixation of mutant individuals, we have the following result.

**Proposition 3.1.**

*Let*

$$H_{N-1}=\sum_{j=1}^{N-1}\frac{1}{N-j}$$

*be the (N−1)-harmonic number. Then the absorption time for a single mutant individual in the Moran process on a complete graph approximates $(N-1)H_{N-1}$ times the absorption time for a single proliferating mutant individual when $p=p_{c}$ and r→+∞, that is, $\lim_{r\to +\infty }\rho (r,p_{c})=(N-1)H_{N-1}$.*

**Remark 1.**

Similarly, we also deduce that $\lim_{r\to +\infty }\tau_{1}^{M}=\lim_{r\to +\infty }T_{1}^{M}=(N-1)H_{N-1}$.

### 3.2. Fixation time for Bernoulli proliferation on complete graphs

As explained in [8], [9] and [10], for i=1,2,…,N−1, the fixation times $T_{i}$ satisfy the equation

(3.10) $$\begin{array}{r} \begin{array}{rl} \Phi_{i}T_{i} & =p_{i,i-1}\Phi_{i-1}T_{i-1}+p_{i,N}\Phi_{N}T_{N}+(1-p_{i,i-1}-p_{i,N})\Phi_{i}T_{i}+\Phi_{i}\\ & =p_{i,i-1}\Phi_{i-1}T_{i-1}+(1-p_{i,i-1}-p_{i,N})\Phi_{i}T_{i}+\Phi_{i}, \end{array} \end{array}$$

as $T_{N}=0$. We use the convention $\Phi_{0}T_{0}=0$ as in [10]. As before, we get

(3.11) $$\begin{array}{r} \begin{array}{rl} T_{i} & =\frac{p_{i,i-1}}{p_{i,i-1}+p_{i,N}}\frac{\Phi_{i-1}}{\Phi_{i}}T_{i-1}+\frac{1}{p_{i,i-1}+p_{i,N}}\frac{\Phi_{i}}{\Phi_{i}}\\ & =\frac{1-\frac{i-1}{N-1}}{1+rp-\frac{i-1}{N-1}}\frac{\Phi_{i-1}}{\Phi_{i}}T_{i-1}+\frac{1}{1+rp-\frac{i-1}{N-1}}\frac{w_{i}}{i}. \end{array} \end{array}$$

Reasoning by recurrence, for i=1,2,…,N−1, we deduce

(3.12) $$T_{i}=\sum_{j=0}^{i-1}\frac{1}{\left[1+rp-\frac{j}{N-1}\right]}\frac{w_{j+1}}{j+1}\frac{\Phi_{j+1}}{\Phi_{i}}\prod_{k=j+1}^{i-1}\frac{1-\frac{k}{N-1}}{1+rp-\frac{k}{N-1}}.$$

Once again, we can compare to classical situation (also described in [10]) where

(3.13) $$T_{i}^{M}=\sum_{j=1}^{N-1}\frac{\Phi_{j}^{M}}{p_{j,j+1}^{M}}\sum_{k=j}^{N-1}\prod_{l=j+1}^{k}\gamma_{l}-\frac{1}{\Phi_{i}^{M}}\sum_{j=1}^{i-1}\frac{\Phi_{j}^{M}}{p_{j,j+1}^{M}}\sum_{k=j}^{i-1}\prod_{l=j+1}^{k}\gamma_{l}.$$

The conventions for sums and products are the same as before. Now observe

$$T_{1}=\frac{r+N-1}{1+rp}=\tau_{1}$$

as only three states (1 and the absorbing states 0 and N) are involved in this computation. As before, the *fixation time rate* is

(3.14) P(r,p)=T1MT1 =(1+rp)N−1r+N−1(∑j=1N−1ΦjMΦ1Mrj+N−jrj(N−j)rN−rjrN−1o)=(1+rp)N−1r+N−1(∑j=1N−1ΦjMrj+N−jrj(N−j)rN−rjmrN−rN−1)=(1+rp)N−1r+N−1(∑j=1N−1rN(rj−1)rj(rN−1)rj+N−jrj(N−j)rN−rjmrN−rN−1),

and hence the *critical fixation time rate* is given by

(3.15) P(r,pc)=rN−1+⋯+r+1rN−2+⋯+r+1N−1r+N−1(∑j=1N−1ΦjMΦ1Mrj+N−jrj(N−j)rN−rjrN−1)=rN−1+⋯+r+1rN−2+⋯+r+1N−1r+N−1(∑j=1N−1rN(rj−1)rj(rN−1)rj+N−jrj(N−j)rN−rjmrN−rN−1).

Observe that for r=1, we get $\rho (1,p_{c})=H_{N-1}$ and $\text{P}(1,p_{c})=N-1$. Finally, comparing equation (3.15) with equation (3.9), as the rate $\Phi_{j}^{M}/\Phi_{1}^{M}$ converges to 1 when r→+∞ (or directly applying proposition 2.1), we immediately deduce the following result:

**Proposition 3.2.**

*The fixation time for a single mutant individual in the Moran process on a complete graph approximates $(N-1)H_{N-1}$ times, the fixation time for a single proliferating mutant individual when $p=p_{c}$ and r→+∞, that is, $\lim_{r\to +\infty }\text{P}(r,p_{c})=(N-1)H_{N-1}$.*

Propositions 3.1 and 3.2 can be graphically visualized in S5 in the supplementary material for some values of N.

### 3.3. Absorption time for Bernoulli proliferation on cycles

For a cycle, we must distinguish when the starting state has one element or more than one element. In the first case, by symmetry, we can assume that the starting state is S={1}. It may evolve to states with zero and three elements as in figure 3a (or also remain at the same state). The non-trivial transition probabilities are given by

$$\begin{array}{rl} p_{1,0} & =\frac{1}{w_{1}},\\ p_{1,3} & =\frac{rp}{w_{1}}\\ \text{and }p_{1,1} & =1-(p_{1,0}+p_{1,3}), \end{array}$$

where $w_{1}=r+N-1$. As depicted in figure 3b, the first equation for the absorption time is given by

(3.16) $$\begin{array}{r} \tau_{1}=p_{1,3}\tau_{3}+(1-p_{1,0}-p_{1,3})\tau_{1}+1, \end{array}$$

as $\tau_{0}=0$, and then

(3.17) $$\tau_{3}=\frac{1+rp}{rp}\tau_{1}-\frac{w_{1}}{rp}.$$

**Figure 3.**
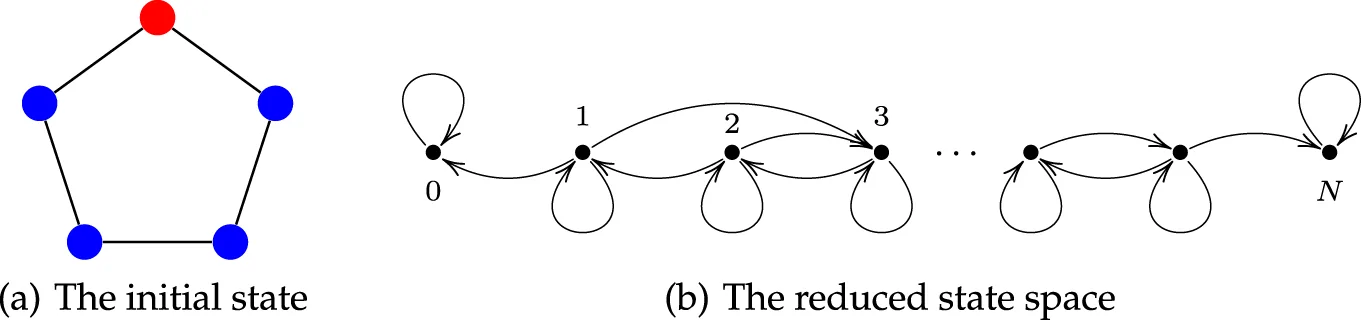
Bernoulli proliferation on a cycle: (a) the initial state S={1} and (b) the reduced state space.

In general, as in the usual Moran model, all individuals of the same type are contiguous. Thus, for i=2,3,…,N−1, the transition probabilities are given by

(3.18) $$\begin{array}{r} \begin{array}{rl} p_{i,i-1} & =\frac{1}{w_{i}},\\ p_{i,i+1} & =\frac{2rp}{w_{i}}\\ \text{and }p_{i,i} & =1-(p_{i,i-1}+p_{i,i+1}), \end{array} \end{array}$$

where $w_{i}=ri+N-i$. The corresponding absorption times are given by the equation

(3.19) $$\begin{array}{rl} \tau_{i} & =p_{i,i-1}\tau_{i-1}+p_{i,i+1}\tau_{i+1}+(1-p_{i,i-1}-p_{i,i+1})\tau_{i}+1. \end{array}$$

As usual (see [10] and [27]), we can solve this system by replacing $\delta_{i}=\tau_{i}-\tau_{i-1}$ to obtain

(3.20) $$\begin{array}{r} \delta_{i+1}=\frac{1}{2rp}\delta_{i}-\frac{w_{i}}{2rp}, \end{array}$$

for i≥2. More generally, we have

(3.21) $$\begin{array}{r} \delta_{i+1}={\left(\frac{1}{2rp}\right)}^{i-1}\delta_{2}-\sum_{j=2}^{i}{\left(\frac{1}{2rp}\right)}^{i-j+1}w_{j}, \end{array}$$

for i=2,3,…,N−1. Then,

(3.22) $$\begin{array}{rl} 0=\tau_{N}-\tau_{0} & =\sum_{i=1}^{N}\delta_{i}=\left(\sum_{i=0}^{N-2}{\left(\frac{1}{2rp}\right)}^{i}\right)\delta_{2}-\sum_{i=2}^{N-1}\sum_{j=2}^{i}{\left(\frac{1}{2rp}\right)}^{i-j+1}w_{j}+\delta_{1}. \end{array}$$

Combining

$$\tau_{2}=\frac{1}{1+2rp}\tau_{1}+\frac{2rp}{1+2rp}\tau_{3}+\frac{w_{2}}{1+2rp}$$

with equation (3.17), we obtain

$$\tau_{2}=(1+\frac{2}{1+2rp})\tau_{1}-\frac{2w_{1}}{1+2rp}+\frac{w_{2}}{1+2rp}$$

and hence

$$\delta_{2}=\tau_{2}-\tau_{1}=\frac{2}{1+2rp}\tau_{1}+\frac{w_{2}-2w_{1}}{1+2rp}=\frac{2}{1+2rp}\tau_{1}-\frac{N}{1+2rp}.$$

Therefore equation (3.22) becomes

(3.23) $$\begin{array}{r} \begin{array}{rl} (1+\frac{2}{1+2rp}\sum_{i=0}^{N-2}(\frac{1}{2rp}{)}^{i})\tau_{1} & =\sum_{i=2}^{N-1}\sum_{j=2}^{i}(\frac{1}{2rp}{)}^{i-j+1}w_{j}\\ & +\frac{N}{1+2rp}\sum_{i=0}^{N-2}(\frac{1}{2rp}{)}^{i}, \end{array} \end{array}$$

and finally we get

(3.24) $$\tau_{1}=\frac{\sum_{i=2}^{N-1}\sum_{j=2}^{i}(\frac{1}{2rp}{)}^{i-j+1}w_{j}+\frac{N}{1+2rp}\sum_{i=0}^{N-2}(\frac{1}{2rp}{)}^{i}}{1+\frac{2}{1+2rp}\sum_{i=0}^{N-2}(\frac{1}{2rp}{)}^{i}}.$$

As for the complete graphs, this value can be compared with the value of the absorption time for one single mutant in the Moran process on the cycle

(3.25) $$\begin{array}{r} \tau_{1}^{M}=r^{N-1}\frac{r-1}{r^{N}-1}\left(\sum_{i=1}^{N-1}\sum_{j=1}^{i}\frac{w_{j}}{r^{i-j+1}}\right)=\frac{r^{N-1}}{r^{N-1}+\cdots +r+1}\left(\sum_{i=1}^{N-1}\sum_{j=1}^{i}\frac{w_{j}}{r^{i-j+1}}\right). \end{array}$$

As is shown in [1], the critical value $p_{c}$ is the only solution of the equation

(3.26) $$\begin{array}{r} \frac{2}{1+2rp}\sum_{i=0}^{N-2}{\left(\frac{1}{2rp}\right)}^{i}=\sum_{i=1}^{N-1}{\left(\frac{1}{r}\right)}^{i}. \end{array}$$

Then the absorption time rate at $p=p_{c}$ becomes, after some simple calculations,

(3.27) $$\begin{array}{r} \rho (r,p_{c})=\frac{\sum_{i=1}^{N-1}\sum_{j=1}^{i}{\left(\frac{1}{r}\right)}^{i-j+1}\left(rj+N-j\right)}{\sum_{i=2}^{N-1}\sum_{j=2}^{i}{\left(\frac{1}{2rp_{c}}\right)}^{i-j+1}\left(rj+N-j\right)+\frac{N}{2}\sum_{i=1}^{N-1}{\left(\frac{1}{r}\right)}^{i}}. \end{array}$$

Observe that, for r=1 and $p_{c}=\frac{1}{2}$, we get

$$\rho (1,p_{c})=\frac{N\sum_{i=1}^{N-1}i}{N\sum_{i=2}^{N-1}(i-1)+\frac{N}{2}(N-1)}=\frac{N}{N-1}.$$

When r→+∞, the situation is now somewhat different from that described in proposition 3.1

**Proposition 3.3.**

*The absorption time rate for a single mutant individual on a cycle graph satisfies the condition*

$$\lim_{r\to +\infty }\rho (r,p_{c})=\frac{2N(N-1)}{\left(N+1)(N-2\right)}.$$

Proof.
From equations (3.24) and (3.25), we deduce $\tau_{1}^{M}/\tau^{1}$ is the product of the following three factors:

(3.28)1+21+2rp(2rp)N−2+⋯+2rp+1(2rp)N−2,(3.29)rN−1rN−1+⋯+r+1,(3.30)O=∑i=1N−1∑j=1iwjri−j+1∑i=2N−1∑j=2i(12rp)i−j+1wj+N1+2rp∑i=0N−2(12rp)i=∑i=2N−1∑j=2irj+N−jri−j+1+∑i=1N−1r+N−1ri∑i=2N−1∑j=2irj+N−j(2rp)i−j+1+N1+2rp(2rp)N−2+⋯+2rp+1(2rp)N−2=D(r)+∑i=1N−1r+N−1riD(2rp)+N1+2rp(2rp)N−2+⋯+2p+1(2rp)N−2.

The two first factors, equations (3.28) and (3.29), converge to 1 as r→+∞. For the third factor, equation (3.30), we have

$$\lim_{r\to +\infty }D(r)=\frac{\left(N+1)(N-2\right)}{2}\;\text{and}\;\lim_{r\to +\infty }D(2rp)=\frac{1}{2p}\frac{\left(N+1)(N-2\right)}{2},$$

so this factor converges to

$$\frac{2pN(N-1)}{\left(N+1)(N-2\right)}.$$

Therefore, we have

$$\lim_{r\to +\infty }\rho (r,p)=\frac{2pN(N-1)}{\left(N+1)(N-2\right)}.$$

Finally, as $p_{c}$ converges to 1 as r→+∞, we deduce

$$\lim_{r\to +\infty }\rho (r,p_{c})=\frac{2N(N-1)}{\left(N+1)(N-2\right)}.$$

**Remark 2.**

From equation (3.25), we also deduce that $\lim_{r\to +\infty }\tau_{1}^{M}=\lim_{r\to +\infty }T_{1}^{M}=\frac{N(N-1)}{2}$.

### 3.4. Fixation time for Bernoulli proliferation on cycles

As before, first we write the equation for the fixation time of one single mutant:

(3.31) $$\begin{array}{rl} \Phi_{1}T_{1} & =p_{1,0}\Phi_{0}T_{0}+p_{1,3}\Phi_{3}T_{3}+(1-p_{1,0}-p_{1,3})\Phi_{1}T_{1}+\Phi_{1}\\ & =p_{1,3}\Phi_{3}T_{3}+(1-p_{1,0}-p_{1,3})\Phi_{1}T_{1}+\Phi_{1}, \end{array}$$

as $\Phi_{0}T_{0}=0$. We deduce

(3.32) $$\begin{array}{r} T_{3}=\frac{\Phi_{1}}{\Phi_{3}}\left(\frac{1+rp}{rp}T_{1}-\frac{w_{1}}{rp}\right). \end{array}$$

In general, to solve the equation

(3.33) $$\begin{array}{rl} \Phi_{i}T_{i} & =p_{i,i-1}\Phi_{i-1}T_{i-1}+p_{i,i+1}\Phi_{i+1}T_{i+1}+(1-p_{i,i-1}-p_{i,i+1})\Phi_{i}T_{i}+\Phi_{i} \end{array}$$

for 2≤i≤N−1, we proceed in two steps:

— First, we replace $z_{i}=\Phi_{i}T_{i}$ to obtain

(3.34) $$\begin{array}{rl} z_{i} & =p_{i,i-1}z_{i-1}+p_{i,i+1}z_{i+1}+(1-p_{i,i-1}-p_{i,i+1})z_{i}+\Phi_{i}. \end{array}$$

— Second, we replace $\delta_{i}=z_{i}-z_{i-1}$ to get

(3.35) $$\delta_{i+1}=\frac{1}{2rp}\delta_{i}-\frac{\Phi_{i}}{p_{i,i+1}}=\frac{1}{2rp}\delta_{i}-\frac{w_{i}\Phi_{i}}{2rp}.$$

As before, we deduce

(3.36) $$\begin{array}{rl} 0=z_{N}-z_{0} & =\sum_{i=1}^{N}\delta_{i}=\left(\sum_{i=0}^{N-2}{\left(\frac{1}{2rp}\right)}^{i}\right)\delta_{2}-\sum_{i=2}^{N-1}\sum_{j=2}^{i}{\left(\frac{1}{2rp}\right)}^{i-j+1}w_{j}\Phi_{j}+\delta_{1}. \end{array}$$

Combining again

$$z_{2}=\frac{1}{1+2rp}z_{1}+\frac{2rp}{1+2rp}z_{3}+\frac{w_{2}\Phi_{2}}{1+2rp}$$

with equation (3.32), we obtain

$$z_{2}=\left(1+\frac{2}{1+2rp}\right)z_{1}-\frac{2w_{1}\Phi_{1}}{1+2rp}+\frac{w_{2}\Phi_{2}}{1+2rp}$$

and hence,

$$\delta_{2}=z_{2}-z_{1}=\frac{2}{1+2rp}z_{1}+\frac{w_{2}\Phi_{2}-2w_{1}\Phi_{1}}{1+2rp}.$$

Therefore, equation (3.36) becomes

(3.37) $$\left(1+\frac{2}{1+2rp}\sum_{i=0}^{N-2}{\left(\frac{1}{2rp}\right)}^{i}\right)z_{1}=\sum_{i=2}^{N-1}\sum_{j=2}^{i}{\left(\frac{1}{2rp}\right)}^{i-j+1}w_{j}\Phi_{j}+\frac{2w_{1}\Phi_{1}-w_{2}\Phi_{2}}{1+2rp}\sum_{i=0}^{N-2}{\left(\frac{1}{2rp}\right)}^{i},$$

and finally we get

(3.38) $$T_{1}=\frac{\sum_{i=2}^{N-1}\sum_{j=2}^{i}{\left(\frac{1}{2rp}\right)}^{i-j+1}w_{j}\frac{\Phi_{j}}{\Phi_{1}}+\frac{2w_{1}-w_{2}\frac{\Phi_{2}}{\Phi_{1}}}{1+2rp}\sum_{i=0}^{N-2}{\left(\frac{1}{2rp}\right)}^{i}}{1+\frac{2}{1+2rp}\sum_{i=0}^{N-2}{\left(\frac{1}{2rp}\right)}^{i}}.$$

Using a similar argument, the identity

$$\begin{array}{r} 0=\sum_{i=1}^{N}\delta_{i}=\left(\sum_{i=0}^{N-1}{\left(\frac{1}{r}\right)}^{i}\right)\delta_{1}-\sum_{i=1}^{N-1}\sum_{j=1}^{i}{\left(\frac{1}{r}\right)}^{i-j+1}w_{j}\Phi_{j}^{M}, \end{array}$$

implies that the fixation time for one single non-proliferating mutant is given by

(3.39) $$T_{1}^{M}=\frac{\delta_{1}}{\Phi_{1}^{M}}=\sum_{i=1}^{N-1}\sum_{j=1}^{i}\frac{w_{j}}{r^{i-j+1}}\Phi_{j}^{M}.$$

For r=1 and $p_{c}=\frac{1}{2}$, the critical fixation rate results in (after using $w_{i}=N$ and $\Phi_{i}^{M}=\Phi_{i}=\frac{i}{N}$, for all 1≤i≤N−1 and some simple algebra)

$$\text{P}(1,p_{c})=\frac{N(N+1)}{\left(N-2)(N+3\right)}.$$

The same argument used before allows us to state the following result

**Proposition 3.4.**

*The fixation time rate for a single mutant individual on a cycle graph satisfies the condition*

$$\lim_{r\to +\infty }\rho (r,p_{c})=\frac{2N(N-1)}{\left(N+1)(N-2\right)}.$$

Propositions 3.3 and 3.4 can be also graphically visualized in S5 in the supplementary material.

### 3.5. Absorption time for Bernoulli proliferation on stars

Using notation from [27], the starting state is denoted by (0,1) or (1,0) depending on whether the mutant is placed in the central vertex (as in figure 4a) or in some peripheral vertex. The transition probabilities depend on the pair (i,ε) where i is the number of peripheral vertices occupied by mutants and ε=0,1 depending on the type of individual that occupies the centre, see figure 4b. We write $p_{i,\varepsilon }^{\pm }$ if the number of mutants increases or decreases. The total reproductive weight depends only on the total number of mutants, that is, j=i+ε, and is denoted $w_{j}$. Thus, if we denote ${\hat{p}}_{i,\varepsilon }^{\pm }$ as the transition probabilities for the embedded jump process (see supplementary material, S2), then

(3.40)τ0,1=p^0,1−τ0,0+p^0,1+τN−1,1+1p0,1−+p0,1+=N−1N−1+rpτ0,0+rpN−1+rpτN−1,1+w1N−1+rpxxxX=w1N−1+rp,(3.41)τ1,0=p^1,0−τ0,0+p^1,0+τ1,1+1p1,0−+p1,0+=1(N−1)rp+1τ0,0+(N−1)rp(N−1)rp+1τ1,1+(N−1)w1(N−1)rp+1=(N−1)rp(N−1)rp+1τ1,1+(N−1)w1(N−1)rp+1(3.42)and τ1,1=p^1,1−τ1,0+p^1,1+τN−1,1+1p1,1−+p1,1+=N−2N−2+rpτ1,0+rpN−2+rpτN−1,1+w2N−2+rpxxxX=N−2N−2+rpτ1,0+w2N−2+rp.

**Figure 4.**
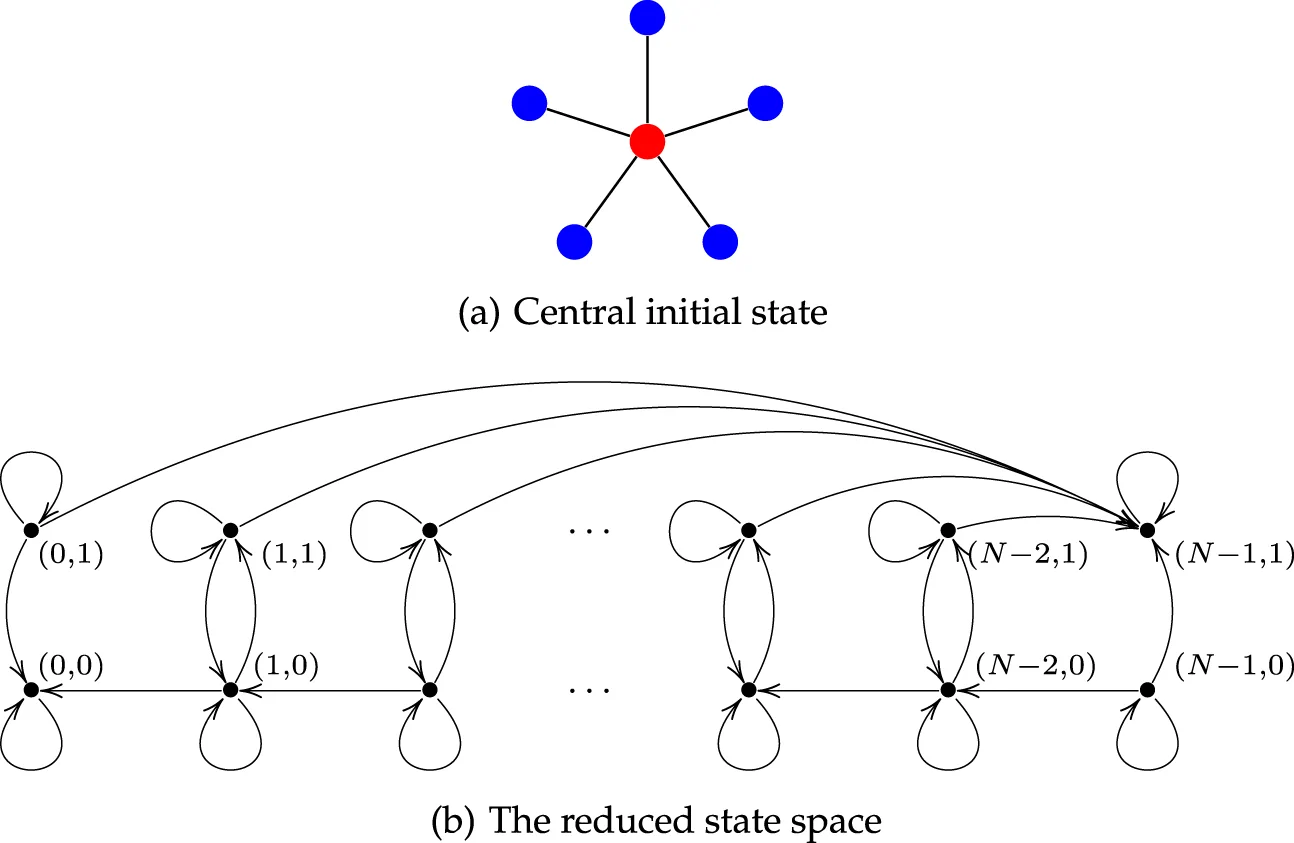
Bernoulli proliferation on a star: (a) central initial state and (b) the reduced state space.

From equations (3.41) and (3.42), we deduce

$$\tau_{1,0}=\frac{(N-1)rpw_{2}}{(N-1)(rp{)}^{2}+rp+N-2}+\frac{(N-1)(rp+N-2)w_{1}}{(N-1)(rp{)}^{2}+rp+N-2}.$$

Adding equation (3.40), the mean absorption time for one single mutant is given by

(3.43) $$\begin{array}{rl} \tau_{1} & =\frac{1}{N}\left(\frac{w_{1}}{N-1+rp}+(N-1{)}^{2}\frac{rpw_{2}+(rp+N-2)w_{1}}{(N-1)(rp{)}^{2}+rp+N-2}\right). \end{array}$$

More generally, we have

(3.44)τi,1=p^i,1−τi,0+p^i,1+τN−1,1+1pi,1−+pi,1+=N−1−iN−1−i+rpτi,0+rpN−1−i+rpτN−1,1+wi+1N−1−i+rp=N−1−iN−1−i+rpτi,0+wi+1N−1−i+rp,(3.45)and τi,0=p^i,0−τi−1,0+p^i,0+τi,1+1pi,0−+pi,0+=1(N−1)rp+1τi−1,0+(N−1)rp(N−1)rp+1τi,1+(N−1)wi((N−1)rp+1)i.

Combining both equations, we obtain the following recurrence formula:

(3.46) $$\begin{array}{r} \begin{array}{rl} \tau_{i,0}= & \;\frac{1}{\left[1-\frac{(N-1)rp}{\left[(N-1)rp+1\right]}\frac{N-1-i}{\left[N-1-i+rp\right]}\right]}(\frac{1}{\left[(N-1)rp+1\right]}\tau_{i-1,0}\\ & +\;\frac{(N-1)rp}{\left[(N-1)rp+1\right]}\frac{w_{i+1}}{\left[N-1-i+rp\right]}\\ & +\;\frac{\left(N-1\right)}{\left[(N-1)rp+1\right]}\frac{w_{i}}{i}). \end{array} \end{array}$$

The absorption time for one single mutant in the Moran process was described in [10]:

(3.47) $$\begin{array}{r} \begin{array}{rl} \tau_{1}^{M}= & \;\frac{1}{N}(\left(\frac{r}{N-1+r}+(N-1{)}^{2}\frac{r}{(N-1)r+1}\right)\frac{1}{D(1)}\sum_{i=2}^{N-1}D(i)E(i)\\ & +\;1+(N-1{)}^{2}\frac{w_{1}}{(N-1)r+1}), \end{array} \end{array}$$

where

$$\begin{array}{rl} D(i) & =1+\frac{N-1}{N-1+r}\sum_{j=i}^{N-2}{\left(\frac{N-1+r}{(N-1)r+1}\right)}^{j-i+1}{\left(\frac{1}{r}\right)}^{j-i+1},\\ E(i) & =\frac{N-1}{r}\sum_{j=1}^{i-1}\frac{(N-1)w_{j}}{((N-1)r+1)j}{\left(\frac{1}{(N-1)r+1}\right)}^{i-j-1}+\frac{(N-1)w_{i}}{(N-i)r}\\ & =\frac{N-1}{r}\sum_{j=1}^{i-1}\frac{\left(N-1)(N-j+rj\right)}{((N-1)r+1)j}{\left(\frac{1}{(N-1)r+1}\right)}^{i-j-1}+\frac{\left(N-1)(N-i+ri\right)}{(N-i)r}. \end{array}$$

**Proposition 3.5.**

*The absorption time for a single mutant individual on a star graph satisfies the condition*

$$\lim_{r\to +\infty }\rho (r,p_{c})=\frac{N}{3N-2}\left[(N-1)\sum_{i=1}^{N-1}H_{i}\right].$$

Proof.
To compute $\lim_{r\to +\infty }\rho (r,p)$, we compute independently the limits of $\tau_{1}$ and $\tau_{1}^{M}$ using equations (3.43) and (3.47). First,

$$\begin{array}{r} \lim_{r\to +\infty }\tau_{1}=\frac{1}{N}\left(\frac{1}{p}+(N-1{)}^{2}\frac{2p+p}{(N-1)p^{2}}\right)=\frac{3N-2}{Np}. \end{array}$$

Second, we compute the limit of each summand in equation (3.47). Since

$$\begin{array}{r} \lim_{r\to +\infty }E(i)=\frac{N-1}{N-i}i \end{array}$$

and

$$\begin{array}{r} \lim_{r\to +\infty }\frac{D(i)}{D(1)}=\lim_{r\to +\infty }\frac{1+\frac{N-1}{N-1+r}\sum_{j=i}^{N-2}{\left(\frac{N-1+r}{(N-1)r+1}\right)}^{j-i+1}{\left(\frac{1}{r}\right)}^{j-i+1}}{1+\frac{N-1}{N-1+r}\sum_{j=1}^{N-2}{\left(\frac{N-1+r}{(N-1)r+1}\right)}^{j}{\left(\frac{1}{r}\right)}^{j}}=1, \end{array}$$

the first summand converges to

$$\sum_{i=2}^{N-1}\frac{N-1}{N-i}i=(N-1)\sum_{i=1}^{N-1}\frac{i}{N-i}-1=(N-1)\sum_{i=1}^{N-1}H_{i}-1,$$

where $\sum_{i=1}^{N-1}H_{i}=NH_{N-1}-(N-1)=N(H_{N-1}-1)+1$. The second summand is equal to 1/N, and finally the third summand converges to $\frac{N-1}{N}$. Therefore, we have

$$\begin{array}{r} \;\lim_{r\to +\infty }\rho (r,p)=\lim_{r\to +\infty }\frac{\tau_{1}^{M}}{\tau_{1}}=\frac{\sum_{i=2}^{N-1}\frac{N-1}{N-i}i+\frac{1}{N}+\frac{N-1}{N}}{\frac{3N-2}{Np}}=p\frac{N}{3N-2}\left[(N-1)\sum_{i=1}^{N-1}H_{i}\right].\; \end{array}$$

Remember finally that $\lim_{r\to +\infty }p_{c}(r)=1$.

**Remark 3.**

From the previous proof, we also deduce that

$$\lim_{r\to +\infty }\tau_{1}^{M}=\lim_{r\to +\infty }T_{1}^{M}=(N-1)\sum_{i=1}^{N-1}H_{i}.$$

Together with remarks 1 and 2, this remark seems to suggest that the limit is related to the *cover time* of the simple random walk (SRW) on the underlying graph (see [28] and [29]); see also S3 in the supplementary material. Both quantities coincide for complete and cycle graphs, although SRW is more efficient in star graphs.

### 3.6. Fixation time for Bernoulli proliferation on stars

Using notation from the previous paragraph, we have

(3.48)z0,1=p^0,1−z0,0+p^0,1+zN−1,1+Φ0,1p0,1−+p0,1+X=w1Φ0,1N−1+rp,(3.49)z1,0=p^1,0−z0,0+p^1,0+z1,1+Φ1,0p1,0−+p1,0+=(N−1)rp(N−1)rp+1z1,1+(N−1)w1Φ1,0(N−1)rp+1(3.50)and z1,1=p^1,1−z1,0+p^1,1+zN−1,1+Φ1,1p1,1−+p1,1+X=N−2N−2+rpz1,0+w2Φ1,1N−2+rp,

where $z_{i,\varepsilon }=T_{i,\varepsilon }\Phi_{i,\varepsilon }$ for any i=0,…,N−1 and any ε=0,1. From equations (3.49) and (3.50), we deduce

$$T_{1,0}=(N-1)\frac{rpw_{2}\frac{\Phi_{1,1}}{\Phi_{1,0}}+(rp+N-2)w_{1}}{(N-1)(rp{)}^{2}+rp+N-2}.$$

Adding equation (3.48), the mean fixation time for one single mutant is given by

(3.51) $$T_{1}=\frac{1}{N}\left(\frac{w_{1}}{N-1+rp}+(N-1{)}^{2}\frac{rpw_{2}\frac{\Phi_{1,1}}{\Phi_{1,0}}+(rp+N-2)w_{1}}{(N-1)(rp{)}^{2}+rp+N-2}\right).$$

More generally, we have

(3.52) (3.52)zi,1=p^i,1−zi,0+p^i,1+zN−1,1+Φi,1pi,1−+pi,1+=N−1−iN−1−i+rpzi,0+wi+1Φi,1N−1−i+rpand zi,0=p^i,0−zi−1,0+p^i,0+zi,1+Φi,0pi,0−+pi,0+(3.53)=1(N−1)rp+1zi−1,0+(N−1)rp(N−1)rp+1zi,1+(N−1)wiΦi,0((N−1)rp+1)i.XXXXXXX

Combining both equations, we obtain a new recurrence formula for the peripheral process:

(3.54) $$\begin{array}{rl} T_{i,0}= & \;\frac{1}{\left[1-\frac{(N-1)rp}{\left[(N-1)rp+1\right]}\frac{N-1-i}{\left[N-1-i+rp\right]}\right]}(\frac{1}{\left[(N-1)rp+1\right]}T_{i-1,0}\frac{\Phi_{i-1,0}}{\Phi_{i,0}}\\ & +\;\frac{(N-1)rp}{\left[(N-1)rp+1\right]}\frac{w_{i+1}}{\left[N-1-i+rp\right]}\frac{\Phi_{i,1}}{\Phi_{i,0}}\\ & +\;\frac{\left(N-1\right)}{\left[(N-1)rp+1\right]}\frac{w_{i}}{i}). \end{array}$$

Once again, combining propositions 2.1 and 3.5, we have

$$\lim_{r\to +\infty }\text{P}(r,p_{c})=\lim_{r\to +\infty }\rho (r,p_{c})=\frac{N}{3N-2}\left[(N-1)\sum_{i=1}^{N-1}H_{i}\right].$$

The behaviour of stars with respect to cliques and cycles can be also graphically visualized in S5 in the supplementary material.

## 4. Absorption and fixation times for binomial proliferation

In this section, we describe the absorption and fixation times for binomial proliferation on a complete graph. We combine the idea used to compute the fixation probability in [1] with the technique used to compute the absorption and fixation times for the usual Moran process in [10]. As before, we suppose the starting mutant is placed at vertex 1. It may evolve to any other state: the embedded jump processes in order N=3,4 are described in figure 5.

**Figure 5.**
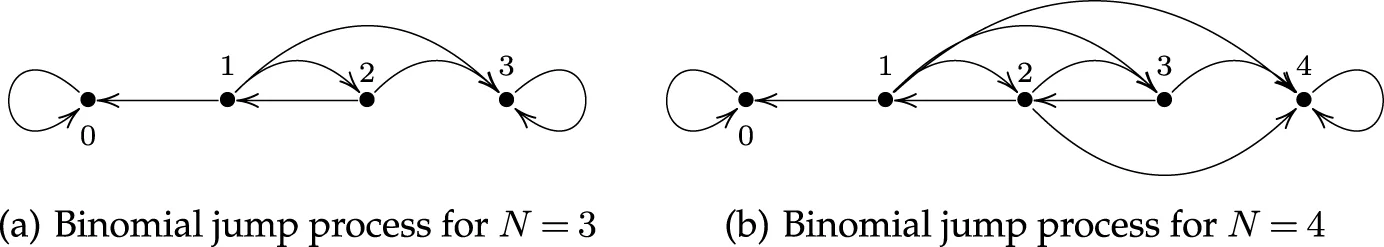
Binomial jump proliferation on a complete graph of (a) order N=3 and (b) order N=4.

The absorption time is given as a solution of the non-homogeneous linear system

(4.1) $$\tau_{i}=p_{i,i-1}\tau_{i-1}+p_{i,i}\tau_{i}+\cdots +p_{i,N}\tau_{N}+1,$$

where i=1,2,…,N−1. If j≥i, the coefficients are

pi,j=riri+N−i(N−ij−i)pj−i(1−p)N−j,

while

$$p_{i,i-1}=\frac{N-i}{ri+N-i}\;\frac{i}{N-1}.$$

We write $\Delta_{i-1,i}=\tau_{i-1}-\tau_{i}$ and $\Delta_{i,j}=\tau_{i}-\tau_{j}$ for j≥i+1. Thus, since $\sum_{j=i-1}^{N}p_{i,j}=1$, we deduce

(4.2) $$p_{i,i-1}\Delta_{i-1,i}=p_{i,i+1}\Delta_{i,i+1}+\cdots +p_{i,N}\Delta_{i,N}-1.$$

As $\Delta_{i,j}=\Delta_{j-1,j}+\Delta_{j-2,j-1}+\cdots +\Delta_{i,i+1}$, we can reorder the terms of equation (4.2) to prove

pi,i−1δi= pi,i+1δi+1+pi,i+2δi+1+pi,i+2δi+2+pi,i+3δi+1+pi,i+3δi+2+pi,i+3δi+3+⋯pi,Nxδi+1+pi,Nxδi+2+pi,Nxδi+3+⋯+pi,NδN−1,

where $\delta_{i}=\Delta_{i-1,i}$ and $\delta_{j}=\Delta_{j-1,j}$ for j≥i+1. Hence, defining

$$w_{i,j}=\frac{1}{p_{i,i-1}}\sum_{k=j}^{N}p_{i,k},$$

we reduce equation (4.1) to the linear system

(4.3) $$\delta_{i}=w_{i,i+1}\delta_{i+1}+w_{i,i+2}\delta_{i+2}+\cdots +w_{i,N-1}\delta_{N-1}+w_{i,N}\delta_{N}-\frac{1}{p_{i,i-1}},$$

where i=1,2,…,N−1. This system can be solved by backward substitution. First, we take

$$\delta_{N-1}=w_{N-1,N}\delta_{N}-\frac{1}{p_{N-1,N-2}}.$$

Substituting in the expression for $\delta_{N-2}$, we get

$$\begin{array}{rl} \delta_{N-2} & =w_{N-2,N-1}\delta_{N-1}+w_{N-2,N}\delta_{N}-\frac{1}{p_{N-2,N-3}}\\ & =(w_{N-2,N-1}w_{N-1,N}+w_{N-2,N})\delta_{N}-\frac{w_{N-2,N-1}}{p_{N-1,N-2}}-\frac{1}{p_{N-2,N-3}}, \end{array}$$

and continue recursively. Notice all possible strictly increasing sequences of numbers from N−2 to N appear in the term involving $\delta_{N}$, while those from N−2 to N−1 (with $w_{N-2,N-2}=1$) appear in the constant terms. In general, let $S_{i,j}$ be the set of strictly increasing sequences (with variable length ℓ) $s\equiv s_{0}<s_{1}<\ldots <s_{\ell }$ of integers such that $s_{0}=i$ and $s_{\ell }=j$ for j≥i, with the convention $w_{j,j}=1$ for all j, and denote $W(s)=w_{s_{0},s_{1}}w_{s_{1},s_{2}}\cdots w_{s_{\ell -1},s_{\ell }}$. We have

(4.4) $$\delta_{i}=\delta_{N}\sum_{s\in S_{i,N}}W(s)-\sum_{j=i}^{N-1}\frac{1}{p_{j,j-1}}\sum_{s\in S_{i,j}}W(s).$$

Arguing similarly to [1], as $\sum_{i=1}^{N}\delta_{i}=0$, we obtain

(4.5) $$\delta_{N}=\frac{\sum_{i=1}^{N-1}\sum_{j=i}^{N-1}\frac{1}{p_{j,j-1}}\sum_{s\in S_{i,j}}W(s)}{\sum_{i=1}^{N}\sum_{s\in S_{i,N}}W(s)}.$$

Finally, using equation (4.4) and the equation above, we get

(4.6) $$\begin{array}{r} \begin{array}{rl} \tau_{1}\;=\;-\delta_{1}\; & =\sum_{j=1}^{N-1}\frac{1}{p_{j,j-1}}\sum_{s\in S_{1,j}}W(s)\\ & -\sum_{\tilde{s}\in S_{1,N}}W(\tilde{s})\frac{\sum_{j=1}^{N-1}\sum_{k=j}^{N-1}\frac{1}{p_{k,k-1}}\sum_{s\in S_{j,k}}W(s)}{\sum_{k=1}^{N}\sum_{s\in S_{k,N}}W(s)}. \end{array} \end{array}$$

Also recall

wi,j=r(N−1)N−i∑k=jN(N−ik−i)pk−i(1−p)N−k.

The same approach can be used to compute the absorption time for the Moran process (or any birth–death process) on a complete graph. In this case, the weight $w_{i,j}=p_{i,i+1}^{M}/p_{i,i-1}^{M}=1/\gamma_{i}$ if j=i+1 and $w_{i,j}=0$ otherwise, and equation (4.3) reduces to

(4.7) $$\delta_{i}=w_{i,i+1}\delta_{i+1}-\frac{1}{p_{i,i-1}^{M}},$$

for i=1,2,…,N−1. Arguing as before, we obtain the following expression:

(4.8) $$\begin{array}{rl} \tau_{1}^{M} & =\sum_{i=1}^{N-1}\frac{1}{p_{i,i-1}^{M}}\prod_{k=1}^{i-1}w_{k,k+1}-\frac{\prod_{l=1}^{N-1}w_{l,l+1}\sum_{i=1}^{N-1}\sum_{j=i}^{N-1}\frac{1}{p_{j,j-1}^{M}}\prod_{k=i}^{j-1}w_{k,k-1}}{1+\sum_{i=1}^{N-1}\sum_{k=i}^{N-1}w_{k,k+1}}. \end{array}$$

However, replacing $\delta_{i}$ by $-\delta_{i}$, equation (4.7) can be replaced by

(4.9) $$\delta_{i+1}=\gamma_{i}\delta_{i}-\frac{1}{p_{i,i+1}^{M}},$$

and now we obtain the following equivalent expression (cf. [5, §4.7]):

(4.10) $$\begin{array}{rl} \tau_{1}^{M} & =\frac{\sum_{i=1}^{N-1}\frac{1}{p_{i,i+1}^{M}}\prod_{k=1}^{i}w_{k,k+1}\sum_{j=i}^{N-1}\prod_{k=j+1}^{N-1}w_{k,k+1}}{\sum_{i=0}^{N-1}\prod_{k=i+1}^{N-1}w_{k,k+1}}. \end{array}$$

The fixation time is now the solution of the non-homogeneous linear system

(4.11) $$\Phi_{i}T_{i}=p_{i,i-1}\Phi_{i-1}T_{i-1}+p_{i,i}\Phi_{i}T_{i}+\cdots +p_{i,N}\Phi_{N}T_{N}+\Phi_{i},$$

where $\Phi_{N}=1$ and $T_{N}=0$. As in the previous computations, writing $z_{i}=\Phi_{i}T_{i}$, we reduce equation (4.11) to the linear system

(4.12) $$z_{i}=p_{i,i-1}z_{i-1}+p_{i,i}z_{i}+\cdots +p_{i,N}z_{N}+\Phi_{i},$$

similar to equation (4.1). This system can be solved using the same method.

## 5. Symmetry and monotonicity

The aim of this section is to prove theorem 2.2 for the Moran process $P^{M}$ and theorem 2.3 for the proliferation processes $P^{\beta }$ with β=B,b. Let P be any process $P^{M}$ or $P^{\beta }$ defined on a connected undirected graph G=(V,E) with fitness r∈(0,+∞) and proliferation parameter p∈(0,1]. The process P identifies to the random walk on the state space S=P(V) with the graph structure given by the transition matrix P. Two states $S\text{ and }S^{\prime }\in S$ are joined by an oriented edge if and only if $p_{S,S^{\prime }}>0$ and this probability becomes the weight of the edge. We call *symmetry* of P any symmetry of the weighted and directed graph S. Denoting P the space of trajectories $\sigma =\{S_{n}{\}}_{n\geq 0}$ and $P_{S_{0}}$ the subspace of trajectories starting at $S_{0}$, the probability measure on $P_{S_{0}}$ is given by

$$P_{S_{0}}[\sigma (0)=S_{0},\ldots ,\sigma (n)=S_{n}]=p_{S_{0},S_{1}}\cdots p_{S_{n-1},S_{n}}.$$

A more complete description can be seen in S1 and S2 in the supplementary material.

### 5.1. G-symmetry

Monotonicity of the expected fixation time in the Moran process will be based on some kind of symmetry. Let us restrict ourselves to the classical case $P=P^{M}$ on the complete graph $G=K_{N}$. Any state S∈P(V) is a clique (of graph G) with i=|S| elements endowed with the subgraph structure. Thus, the symmetries of G become symmetries of P reducing it to the random walk on the quotient graph $K_{N}$, which is described in figure 6.

**Figure 6.**
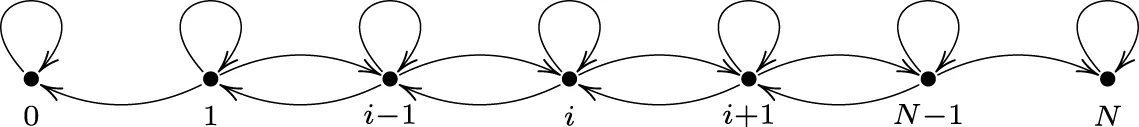
The reduced state space $K_{N}$ for the Moran process $P^{M}$.

The fixation probability $\Phi_{S_{0}}$ is equal to the fixation probability $\Phi_{\left|S_{0}\right|}$ of the reduced model. We also know that $P^{M}$ can be replaced by the embedded jump process ${\hat{P}}^{M}$ (supplementary material, S2). It is easy to see that the reduced process of ${\hat{P}}^{M}$ is the embedded jump process of the reduced process of $P^{M}$ with forward probability $\frac{r}{r+1}$ and backward probability $\frac{1}{r+1}$. Its state space ${\hat{K}}_{N}$ is also shown in figure 7. However, to compute the expected fixation time, some care must be taken. If the number of non-trivial transitions of a trajectory $\sigma \in P_{S_{0}}$ is equal to n, then the length of σ (on average) is given by

(5.1) $$\tau_{S_{0}}(\sigma )=n+\sum_{i=0}^{n-1}\frac{p_{S_{i},S_{i}}}{1-p_{S_{i},S_{i}}}=n+\sum_{i=0}^{n-1}\left(\frac{1}{1-p_{S_{i},S_{i}}}-1\right)=\sum_{i=0}^{n-1}\frac{1}{1-p_{S_{i},S_{i}}},$$

where the sojourn time in each state $S_{i}$ does not depend on $S_{i}$, but only on $\left|S_{i}\right|$.

**Figure 7.**
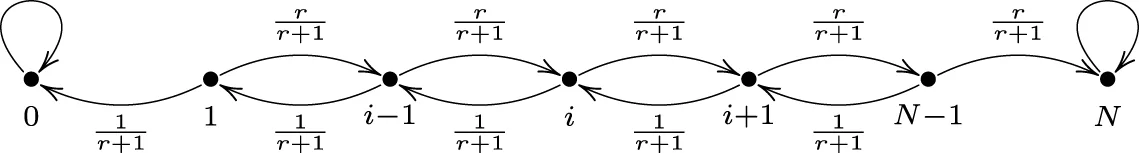
The state space ${\hat{K}}_{N}$ for the embedded jump process ${\hat{P}}^{M}$ on a complete graph $G=K_{N}$.

Now, we can observe that the weighted graph $K_{N}$ has a global symmetry, which we call *G-symmetry*, when we replace r with 1/r. Equivalently, this symmetry applies to the weighted graph ${\hat{K}}_{N}$ and the probabilities

(5.2) $$p_{i,i}=p_{i,i}(r)=\frac{ri}{ri+N-i}.\frac{i-1}{N-1}+\frac{N-i}{ri+N-i}.\frac{N-i-1}{N-1}$$

(or in the same way the sojourn times $1/1-p_{i,i}$) decorating the vertices of ${\hat{K}}_{N}$. That is, $p_{i,i}(\frac{1}{r})=p_{N-i,N-i}(r)$ for all i=1,…,N−1 and all r>0.

As we explain in S2 in the supplementary material, conditioning to the fixation event is equivalent to applying the *Doob transform* to the process P. A detailed definition can be found in [29, ch.17] and also in the above cited in the supplementary material. From proposition S1 in the supplementary material, the embedded jump process of the Doob transform coincides with the Doob transform of the embedded jump process. Now, using G-symmetry, we retrieve the symmetry between the expected fixation time for i mutant individuals and the expected extinction time for N−i resident individuals, formulated by Maruyama [11] and Maruyama & Kimura in [12] (see also [6,8,13]).

We also see that the expected fixation times of mutants of fitness r and 1/r are identical, an observation which is derived from the Maruyama–Kimura symmetry in [7]. However, we should note that the results from [6] and [7] are more general and involve the whole fixation time distribution.

The same construction applies when G is a cycle graph, exhibiting a global G-symmetry for all essential states in the sense of [30] (figure 8). They form the communicating class of 1. But below we describe two examples where this G-symmetry is broken.

**Figure 8.**
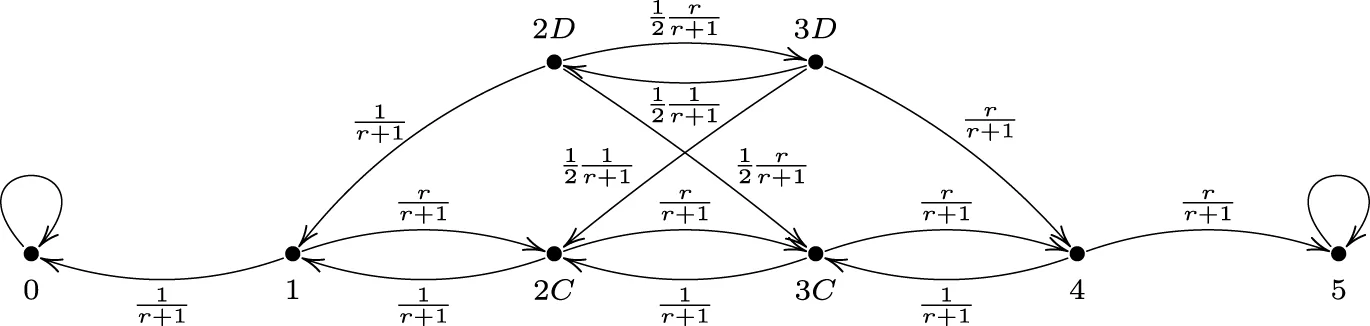
The reduced state space of ${\hat{P}}^{M}$ on the cycle of order N=5. States marked with C are connected and essential, and those marked with D are disconnected and inessential.

### 5.2. Two examples

Let $L_{3}$ be the line graph with three vertices, that is, the star graph $K_{1,2}$. Keeping in mind the notation used in §3, it is easy to describe the reduced state space $\hat{S}$ of the embedded jump process ${\hat{P}}^{M}$, as drawn in figure 9.

**Figure 9.**
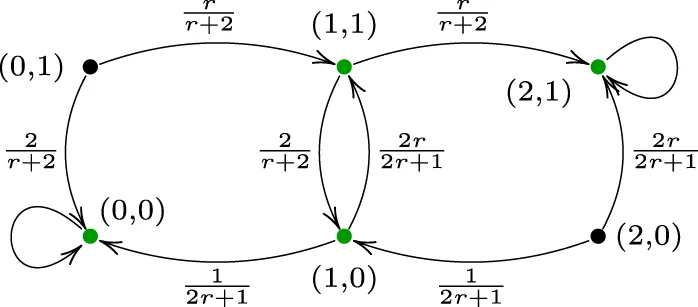
The reduced state space of ${\hat{P}}^{M}$ on the line $L_{3}$.

Obviously, the central vertex and the two peripheral vertices show different behaviour, and G-symmetry does not even make sense. Indeed, although the transition and sojourn probabilities are symmetric, the complementary states (0,1) and (2,0) do not belong to the same communicating class. However, the subgraph of $\hat{S}$ determined by the green-coloured states admits a global G-symmetry since the sojourn probabilities $\frac{3}{2}.\frac{1}{r+2}$ and $\frac{3}{2}.\frac{r}{2r+1}$ at the states (1,0) and (1,1) are also exchanged when we replace r with 1/r. Thus, the peripheral vertices of $L_{3}$ have the same expected fixation time, but the mean fixation time is not preserved by this symmetry.

Let us next consider the octahedron $G=O_{6}$, that is, the four-regular graph of order 6. According to the circulation theorem of [2], the Moran process on $O_{6}$ is equivalent to the Moran process on $K_{6}$. This means that the number of elements |S| of states S∈S performs a random walk on the integer interval [0,N] with absorbing states 0 and N and forward bias r. Indeed, using the symmetries of G, the state space of ${\hat{P}}^{M}$ could be reduced to the graph ${\hat{K}}_{6}$ (figure 7). However, this argument is only valid for the computation of the fixation probability since the symmetries of G are not symmetries of $\hat{S}$. Distinguishing four central vertices from two peripheral vertices (as before for the star $K_{1,2}$), we obtain an intermediate state space ${\hat{S}}_{1}$ for ${\hat{P}}^{M}$, drawn in figure 10.

**Figure 10.**
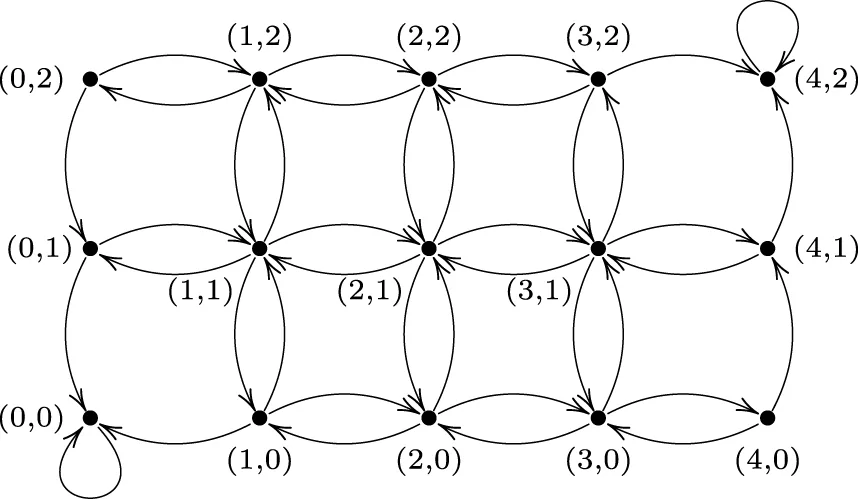
A reduction of the state space of ${\hat{P}}^{M}$ on the octahedron $G=O_{6}$.

The symmetries of G allow us to identify all states in $\hat{S}$ or ${\hat{S}}_{1}$ with a single vertex. The same happens for all states with five vertices in $\hat{S}$ or ${\hat{S}}_{1}$. However, for states with two vertices, we retrieve the same connectedness phenomenon as for the cycle, although reduced states 2C and 2D are both essential. For states with three vertices, we can find two different subgraph structures induced by the graph structure of G, the line and the cycle, that we denote 3L and 3T. For states with four vertices, two different subgraph structures are also found: two triangles with a common face 4T and a cycle 4C. As mentioned above, for states with five vertices, only a pyramidal structure is induced. For computing the fixation probability of any state, the only thing that matters is its number of elements. However, the subgraph structure is important to computing its expected fixation time. The sojourn time in state 3T is higher than in 3L since their probabilities are related by $p_{3T,3T}=\frac{3}{2}p_{3L,3L}$. The reduced state space is shown in figure 11.

**Figure 11.**
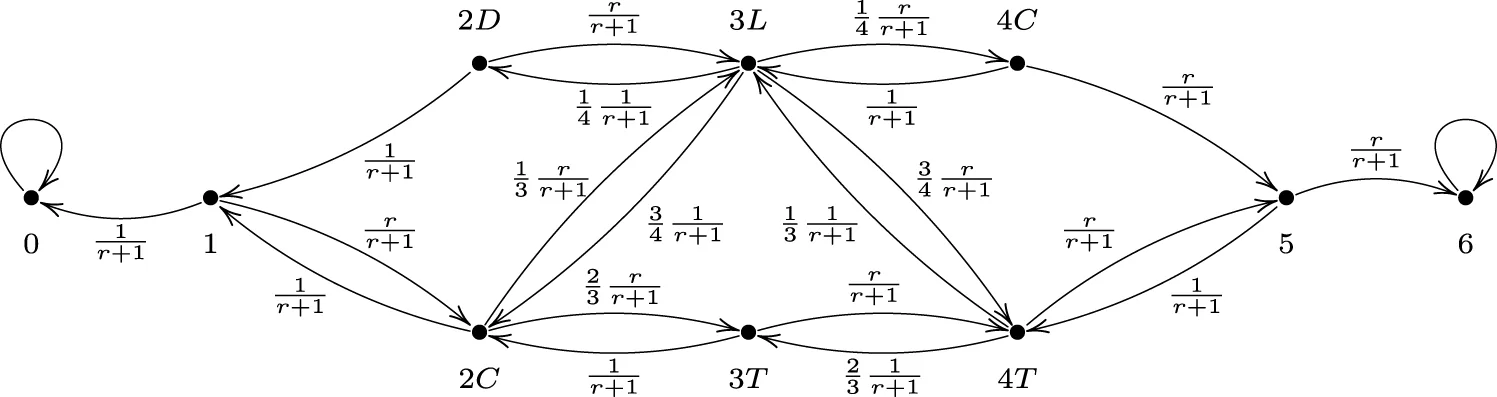
The reduced state space of ${\hat{P}}^{M}$ on the octahedron $G=O_{6}$.

Observe that 3T evolves forward necessarily to 4T, whereas 3L evolves forward to 4C with probability 1/3 and to 4T with probability 2/3. In the other direction, 4T evolves backward equiprobably to 3L or 3T. In summary, there is no G-symmetry, since we need to distinguish the different subgraph structures induced in states of order 3 and 4.

### 5.3. Monotonicity in r

A consequence of G-symmetry for complete and cycle graphs is the fact that the maximum fixation time corresponds to the neutral drift r=1. The two previous examples, line and octahedron, show that this symmetry can partially persist, but it is globally broken (except in the two cases just mentioned). This implies a shift in the fitness value at which the maximum fixation time is reached, showing that the symmetry in figure 1b is only apparent.

Proof of theorem 2.2.
For any graph G of order N, there are only a finite number of states in which the G-symmetry on $K_{N}$ can be broken. Therefore, the fitness value at which the maximum fixation time is reached can only be shifted finitely many times. For each vertex v∈V, as a rational function has finitely many local maxima, there is a fitness value r(v)>0 such that the expected fixation time $T_{\left\{v\right\}}^{M}(r)$ decreases for r≥r(v).

The same argument applies for the mean fixation time $T_{1}^{M}$ (as convex combination of functions $T_{\left\{v\right\}}^{M}$) and the mean absorption time $\tau_{1}^{M}$ (as convex combination of the mean fixation and extinction times according to supplementary material, S1). Obviously our argument does not allow us to say whether the maximum shifts to the right or left, or to comment on the amplitude of the shift. For example, in the line $L_{3}$, the maximum is reached at r=1.4253, whereas r=0.9997 in the octahedron. In fact, for the graphs of order 6, the fitness value where the maximum is reached ranges between r=0.9991 and r=1.2559.

### 5.4. Graphical model

To prove theorem 2.3 about the mononicity of expected fixation time depending on the proliferation parameter, we now consider the proliferation process $P=P^{\beta }$ with β=B,b. Let us recall that Moran and proliferation processes can be obtained from a graphical representation [1], which is inspired by Harris’s representation for contact processes [31] (see also [32]). This realization is done on the set G×[0,+∞) and we start by taking two independent Poisson distributions

$$\Pi_{\varepsilon }(v)=\{\tau_{i}^{\varepsilon }(v):i\in N^{*}\}$$

on [0,+∞), with rate r for ε=1 and rate 1 for ε=2. Each distribution determines a set of coloured marks

$$M_{\varepsilon }=\{(v,\tau )\in V\times [0,+\infty ):\tau \in \Pi_{\varepsilon }(v)\},$$

red for ε=1 and blue for ε=2. Next, we add two random sets of red and blue arrows. For any (v,τ)∈V×[0,+∞), we denote A(v,τ)⊂E×{v} the set of edges with origin v. The set $A_{1}$ of red arrows is defined as follows:

— For the Moran process, an element of A(v,τ) is chosen at random if $(v,\tau )\in M_{1}$.
— For Bernoulli proliferation, we have $A_{1}(v,\tau )=A(v,\tau )$ with probability p and $A_{1}(v,\tau )=\emptyset$ with probability 1−p.
— For binomial proliferation, each element of $A_{1}(v,\tau )$ is chosen independently at random in A(v,τ) with probability p.

To define $A_{2}$, in a manner similar to that which occurs in the Moran process, a unique element of A(v,τ) is chosen at random if $(v,\tau )\in M_{2}$.

From this graphical model, we obtain a continuous-time Markov chain $\{X_{t}{\}}_{t\in [0,+\infty )}$ which will realize the discrete-time Markov chain $\{S_{n}{\}}_{n\in N}$ provided by P. From now on, we shall not distinguish between the expected absorption and fixation times of both types of processes, since they differ only in a factor that does not affect the inequalities that interest us. In fact, it will be convenient to consider only continuous-time processes, even if the results are stated for discrete-time processes. If $P=P^{\beta }$, we write $\left\{X_{t}^{\beta }\right\}$ if we need to specify it. Ignoring blue marks and arrows, we obtain another continuous-time Markov chain, which we denote by $\left\{{\bar{X}}_{t}\right\}$ and call the *red process*. Up to scale, this process is uniquely determined by the underlying graph.

Processes with the same fitness, but associated with different proliferation parameters $0<p^{\prime }<p$ can be coupled taking the same random set of red and blue marks, but randomly deleting red arrows (see supplementary material, S3 for details). Obviously, if the overall processes $\left\{X_{t}\right\}$ and $\left\{X_{t}^{\prime }\right\}$ are coupled, then the red processes $\left\{{\bar{X}}_{t}\right\}$ and $\left\{{\bar{X}}_{t}^{\prime }\right\}$ are also coupled.

For the red process, under Moran updating and conditioning to an initial state ${\bar{X}}_{0}=S_{0}=\{v\}$, we define the first sojourn time as

$${\bar{t}}_{1}=\inf\{t\in [0,+\infty ):{\bar{X}}_{t}\neq {\bar{X}}_{0}\}.$$

Recursively, if ${\bar{t}}_{n}<+\infty$, we define

$${\bar{t}}_{n+1}=\inf\left\{t\in [{\bar{t}}_{n},+\infty ):{\bar{X}}_{t}\neq {\bar{X}}_{{\bar{t}}_{n}}\right\}.$$

All sojourn times

$${\bar{s}}_{n}={\bar{t}}_{n}-{\bar{t}}_{n-1}$$

follow exponential distributions, but the parameters depend on the states $S_{n-1}$. Given the random variable

$$\bar{n}=\sup\{n\geq 1:{\bar{t}}_{n}<+\infty \},$$

the expected fixation time ${\bar{T}}_{S_{0}}$ is the expectation of the random variable

(5.3) $${\bar{t}}_{\bar{n}}=\sum_{n=1}^{\infty }{\bar{t}}_{n}{𝟙}_{\left\{\bar{n}=n\right\}},$$

where ${𝟙}_{\left\{\bar{n}=n\right\}}$ is the characteristic function of the event $\left\{\bar{n}=n\right\}$. We have already described the mean fixation time ${\bar{T}}_{1}$ of the (neutral) red process for complete, cycle and star graphs, as it coincides with the limit $\lim_{r\to +\infty }\;T_{1}^{M}$ analytically computed in remarks 1, 2, and 3. As observed in S3 in the supplementary material, if the sojourn times ${\bar{s}}_{n}$ are identically distributed, then Wald’s first lemma implies that

(5.4) $${\bar{T}}_{S_{0}}=E[{\bar{t}}_{\bar{n}}]=E[{\bar{t}}_{1}]\;E[\bar{n}].$$

More generally, returning to the overall process $\left\{X_{t}\right\}$, we can also define the first sojourn time

$$t_{1}=\inf\{t\in [0,+\infty ):X_{t}\neq X_{0}\},$$

and recursively,

$$t_{n+1}=\inf\{t\in [t_{n},+\infty ):X_{t}\neq X_{t_{n}}\},$$

if $t_{n}<+\infty$. The conditional distribution of $t_{1}$ given by $X_{0}=S_{0}=\{v\}$ is exponential with parameter $\lambda (S_{0})$. Similarly,

$$s_{n}=t_{n}-t_{n-1}$$

follows an exponential distribution of parameter $\lambda (S_{n-1})$ when we condition to the state $S_{n-1}=X_{t_{n-1}}$ in S. Thus, if $\left\{S_{n}\right\}$ denotes the embedded jump chain associated with $\left\{X_{t}\right\}$ and if we condition to $X_{0}=S_{0},\ldots ,X_{t_{n-1}}=S_{n-1}$, the variable

$$t_{n}=s_{1}+\cdots +s_{n}$$

has a hypoexponential distribution with parameters $\lambda (S_{0}),\ldots ,\lambda (S_{n-1})$. This description should be compared to that of [5, §4.8]). Defining

$$n=\sup\{n\geq 1:t_{n}<+\infty \},$$

then equation (5.3) becomes

$$t_{n}=\sum_{n=1}^{\infty }t_{n}{𝟙}_{\left\{n=n\right\}},$$

and equation (5.4) can be reformulated as follows (see again supplementary material, S3): for any state $S_{0}=\{v\}$, if the sojourn times $s_{n}$ are identically distributed, then the expected fixation time is given by

(5.5) $$T_{S_{0}}=E[t_{n}|F_{S_{0}}]=E[t_{1}|F_{S_{0}}]E[n|F_{S_{0}}],$$

where $F_{S_{0}}$ is the fixation event. Nevertheless, this approach to obtain monotonicity in r and p depends strongly on the hypothesis about the identical distribution of sojourn times. Furthermore, even under this constraint, it is not possible to prove monotonicity in r, not even for the expected value of the first sojourn time $E[{\bar{t}}_{1}]$ or $E[t_{1}|F_{S_{0}}]$.

### 5.5. Monotonicity in p

Let $P^{\beta }$ be the proliferation process on G where β=B,b. As before under Moran updating, we can now define random variables $t_{1}^{\beta }$, $t_{n}^{\beta }$ and $t_{n}^{\beta }$ associated with the continuous-time Markov chain $\left\{X_{t}^{\beta }\right\}$ under β proliferation updating. The random variables associated with the corresponding red process are denoted ${\bar{t}}_{1}^{\beta }$, ${\bar{t}}_{n}^{\beta }$ and ${\bar{t}}_{\bar{n}}^{\beta }$. As before, we choose a vertex v and take $S_{0}=\{v\}$ as initial state.

Given any fitness value r>0, if the proliferation parameters $0<p^{\prime }\leq p$, then

(5.6) $${\bar{t}}_{n}^{\beta }(p^{\prime })\geq {\bar{t}}_{n}^{\beta }(p)$$

for any pair of coupled trajectories. Therefore,

(5.7) $$E[{\bar{t}}_{n}^{\beta }(p^{\prime })]\geq E[{\bar{t}}_{n}^{\beta }(p)],$$

and then

(5.8) $$E[{\bar{t}}_{{\bar{n}}^{\prime }}^{\beta }(p^{\prime })]\geq E[{\bar{t}}_{\bar{n}}^{\beta }(p)].$$

In the overall process, the blue marks and arrows modify the value of probabilities and times, but they do not modify the inequality after conditioning.

Proof of theorem 2.3.
Replacing the red process by the overall process and translating the conditioning by using fixation probabilities (as we describe in the supplementary material, S2), we deduce

(5.9) $$\begin{array}{r} \begin{array}{rl} T_{S_{0}}^{\beta }(r,p^{\prime })= & \frac{E[t_{n^{\prime }}^{\beta }(p^{\prime })]}{\Phi_{S_{0}}^{\beta }(r,p^{\prime })}\\ & \geq \frac{E[t_{n^{\prime }}^{\beta }(p^{\prime })]}{\Phi_{S_{0}}^{\beta }(r,p)}\\ & \geq \frac{E[t_{n}^{\beta }]}{\Phi_{S_{0}}^{\beta }(r,p)}=T_{S_{0}}^{\beta }(r,p), \end{array} \end{array}$$

proving the result.

## 6. Critical proliferation

In this section, we shall prove theorem 2.4, that is, the existence of a second critical value of the proliferation parameter $p_{t}^{\beta }$ such that β proliferation is advantageous in term of fixation time if $p\geq p_{t}^{\beta }$ and disadvantageous if $p\leq p_{t}^{\beta }$. The proof will be immediately derived from three lemmas. The first lemma is a corollary of theorem 2.3.

**Lemma 6.1.**

*For any graph G and any fitness value r>0, the mean fixation rate ${\text{P}}^{\beta }(r,p)$ is monotonically increasing in p.*

Proof.
The mean fixation rate

$${\text{P}}^{\beta }(r,p)=\frac{T_{1}^{M}(r)}{T_{1}^{\beta }(r,p)}$$

is obviously monotonically increasing in p since the mean fixation time $T_{1}^{\beta }(r,p)$ is monotonically decreasing in p according to theorem 2.3.

From the previous remark, we know that both limits $\lim_{p\to 0}T_{1}^{\beta }(r,p)$ and $\lim_{p\to 0}{\text{P}}^{\beta }(r,p)$ always exist.

**Lemma 6.2.**

*For any graph G and any fitness value r>0, $\lim_{p\to 0}{\text{P}}^{\beta }(r,p)<1$.*

Proof.
For each vertex v∈V, there exists p>0 small enough to assure that the expected value of the sojourn time in the initial state $S_{0}=\{v\}$ (which depends on p when β=B, but also on d(v) when β=b) is greater than the mean fixation time $T_{1}^{M}(r)$. As V is finite, we deduce that all expected fixation times $T_{\left\{v\right\}}^{\beta }(r,p)>T_{1}^{M}(r)$ for p small enough. It follows that

$$\lim_{p\to 0}T_{1}^{\beta }(r,p)>T_{1}^{M}(r),$$

and then

$$\lim_{p\to 0}{\text{P}}^{\beta }(r,p)<1,$$

as we wanted.

**Lemma 6.3.**

*If $G\neq K_{2}$, then ${\text{P}}^{\beta }(r,1)\geq 1.$*

Proof.
First, observe that the Moran process $P^{M}$ and the proliferation process $P^{\beta }$ can be coupled if p=1. Furthermore, if $G\neq K_{2}$, the number of red arrows must be ≥2 for some vertex v∈V. This implies $T_{\left\{v\right\}}^{\beta }(r,1)\leq T_{\left\{v\right\}}^{M}(r)$ and then

$${\text{P}}^{\beta }(r,1)=\frac{T_{1}^{M}(r)}{T_{1}^{\beta }(r,1)}\geq 1,$$

as we asserted.

Proof of theorem 2.4.
For any graph $G\neq K_{2}$ and any fitness value r>0, the fixation time rate ${\text{P}}^{\beta }(r,p)<1$ for p small enough from lemma 6.2 and ${\text{P}}^{\beta }(r,1)\geq 1$ from lemma 6.3. From lemma 6.1, there exists a unique proliferation value $p_{t}=p_{t}^{\beta }(r)\in (0,1]$ such that ${\text{P}}^{\beta }(r,p_{t})=1$. For $p<p_{t}$, the rate ${\text{P}}^{\beta }(r,p_{t})<1$ and the proliferation is disadvantageous, and for $p>p_{t}$ the rate ${\text{P}}^{\beta }(r,p_{t})>1$ and the proliferation is advantageous.

## 7. Conclusion

*Proliferation processes* were introduced in [1] as a realistic method for increasing the fixation probability of mutant individuals in populations structured on graphs. However, somewhat surprisingly, we found that there is always a value of the proliferation parameter depending on the graph structure in which the fixation probability was the same. For lower values, proliferation is disadvantageous; only for higher values is it advantageous. However, it can be expected that the occupation of neighbouring nodes following Bernoulli or binomial draws will always be advantageous in terms of (mean) fixation time.

This idea was initially supported by the analytical computation of mean absorption and fixation times for Bernoulli proliferation on complete, cycle and star graphs in §3 and binomial proliferation on complete graphs in §4. We have applied methods similar to those used in [8], [9] and [10] to solve the equations formulated from the transition matrix of the Moran process. Exact computations of mean fixation times for all graphs of order 6 (included in supplementary material) raised two questions about this process: first the apparent symmetry between the mean fixation time for mutants with fitness r and 1/r and second the monotonicity of the mean fixation time when r>1. As observed by Hathcock & Strogatz in [7], this symmetry can be derived from Maruyama–Kimura symmetry, introduced in [11] and [12] and rediscovered in [8], [6] and [13]. We derive this symmetry from some symmetry of the process, which we call *G-symmetry*, only observed in cliques and cycles. As a consequence, the maximum fixation time for these graphs is reached when r=1, but the breaking of the G-symmetry implies a shift to the right or the left. Our method does not allow us to compute the displacement, nor predict its direction. We only know that the mean fixation time is monotonically decreasing from this value. It would be interesting to find another method that explains this shift and, more precisely, why the values found by numerical methods for regular graphs of order 6 are less than 1. This issue ties in with another related question, which is not addressed in this paper, concerning the existence of transitions between different temporal regimes, similar to the amplifier/suppressor transitions observed for the fixation probability in [17,25].

Contrary to the initial idea, supported by analytical calculations for cliques, cycles and stars, we have proven that there is a second critical value $p_{t}$ such that proliferation is no longer advantageous for $p\leq p_{t}$. As in [1], we have resorted to a graphical model based on Harris’s graphical method [31] and reduced the analysis to a ‘non-backward’ process that we have called *red process*. This method has allowed us to demonstrate that the mean fixation time decreases monotonically when the proliferation parameter increases, providing the second critical value, but does not explain the entire dynamics of the process. An open question is the existence of graph structures where proliferation is advantageous in probability and disadvantageous in time in the intermediate regime. Another issue to be studied in the future is the relation between the fixation time for the (neutral) red process and the cover time for the SRW on the underlying graph.

Our main contribution in this work has been to clarify the role of symmetries of the Moran process for computing fixation time in a population structured on a graph. Unlike what happens with the fixation probability, the Maruyama–Kimura symmetry and the symmetry for mutants with fitness r and 1/r depend on the graph structure induced on each state, justifying some asymmetry in all graphs except cliques and cycles. The application of Harris’s graphical method to the study of Moran and proliferation processes is promising, but we have only used it to study the dependence of the mean fixation time on the proliferation parameter. It is possible that the combination of these methods with others, whether classical (such as those used by Durrett, Grimmett and their co-authors [33–36] in the study of contact processes) or more recent (such as the martingale method proposed by Monk & van Schaik in [37] and [38]), will allow progress in the study of the absorption and fixation times of evolutionary processes on graphs. We are interested in processes based on evolutionary Markov chains [39,40] and those such as DB, IM or PC obtained by changing the updating rules [14,15]. A reasonable comparison of proliferation mechanisms with these processes, inspired by the voter model, will require the development of strategies to ensure that mutants, such as stem cells or prions, instantly take over subpopulations of resident cells.

## Supplementary Material



## Data Availability

Exact computation of absorption and fixation times for all graphs of order 6 and for cliques, cycles and star graphs of order 3, 10, 20 and 30 is accessible in http://github.com/rojizo/proliferationTimes. It is a modification of the code used to compute the fixation probability for the Moran process [25] and for proliferation processes in [1]. The code is permanently accessible on Zenodo [41]. Supplementary material is available online [42].
